# Gelatin Methacryloyl Hydrogel Encapsulating CiMECs-Derived Extracellular Vesicles Ameliorates Lactation Function via Alleviating Mammary Oxidative Stress

**DOI:** 10.3390/antiox15091115

**Published:** 2026-09-04

**Authors:** Guodong Wang, Jiawen Duan, Longfei Sun, Tao Xu, Jianwei Chen, Aihao Xu, Quanhui Liu, Mengqin Qin, Shouyu Huo, Weiqing Li, Xiaozhen Li, Quanqing Zou, Prasanna Kallingappa, Dandan Zhang, Ben Huang

**Affiliations:** 1 Guangxi Zhuang Autonomous Region Engineering Research Center for 3D Printing in Smart Biomanufacturing and Application, Guangxi Academy of Medical Sciences, Nanning 530021, China; guodongwangbio@163.com (G.W.); duanjiawenbio@163.com (J.D.); sunmulinxi@163.com (L.S.); chenjw@tsinghua-sz.org (J.C.); xuaihao1231@163.com (A.X.); qrmyylxz@163.com (X.L.); zouquanqing@136.com (Q.Z.); 2 Center for Bio-Intelligent Manufacturing and Living Matter Bioprinting, Research Institute of Tsinghua University in Shenzhen, Tsinghua University, Shenzhen 518057, China; xut@tsinghua-sz.org; 3 Tsinghua Shenzhen International Graduate School, Tsinghua University, Shenzhen 518055, China; 4 Guangxi Key Laboratory of Electrochemical Energy Materials, School of Chemistry and Chemical Engineering, Guangxi University, Nanning 530004, China; 5 School of Animal Science and Technology, Guangxi University, Nanning 530005, China; liuquanhui1993@163.com (Q.L.); qmq199511@163.com (M.Q.); 15363439546@163.com (S.H.); 15887560958@163.com (W.L.); 6 Vernon Jansen Unit, Faculty of Medical and Health Sciences, The University of Auckland, Auckland 1010, New Zealand; p.kallingappa@auckland.ac.nz

**Keywords:** oxidative stress, extracellular vesicles, gelatin methacryloyl, postpartum hypogalactia, redox homeostasis

## Abstract

**Background**: Postpartum hypogalactia is a prevalent obstetric complication worldwide, closely associated with excessive oxidative stress and impaired antioxidant defense in mammary tissue. Current hormone-based therapies carry endocrine disruption risks, while natural antioxidant bioactive agents such as extracellular vesicles (EVs) are largely limited by rapid in vivo clearance and poor tissue retention. **Methods**: We constructed an injectable gelatin methacryloyl (GelMA) hydrogel system to encapsulate chemically induced mammary epithelial cell-derived EVs (CiMECs-EVs) and systematically evaluated their antioxidant and lactogenic activities via multi-omics analysis, cellular functional assays and a bromocriptine-induced murine hypogalactia model. **Results**: CiMECs-EVs induced a functional mammary epithelial-like phenotype in fibroblasts in a dose-dependent manner with functional cargo enriched in glutathione metabolism and redox-regulatory miRNAs. The GelMA matrix protected EV integrity and enabled sustained release, and the composite system significantly ameliorated mammary duct structure and lactation function in vivo with specific mammary tropism and no systemic toxicity, outperforming free EV treatment. **Conclusions**: This study presents a safe protein biomacromolecule-based antioxidant delivery platform that effectively restores mammary redox balance and antioxidant defenses, providing a promising non-hormonal therapeutic strategy for postpartum hypogalactia.

## 1. Introduction

According to the 2023 World Health Organization (WHO) Progress Report on Improving Maternal and Newborn Health and Reducing Stillbirths, maternal and newborn health remains a global public health priority, with an estimated 4.5 million combined deaths (290,000 maternal deaths, 1.9 million stillbirths, and 2.3 million newborn deaths) annually—most of which are preventable. Notably, postpartum hypogalactia’s link to suboptimal breastfeeding practices and adverse maternal–neonatal outcomes is well established: inadequate milk production contributes to early breastfeeding cessation (within 6 months) and increases the risk of infant malnutrition, particularly in low- and middle-income countries, where 75% of maternal and newborn deaths occur [1]. A large-scale multicenter cohort study conducted in China, encompassing 120,000 postpartum women across 23 provinces, reported an overall incidence of postpartum hypogalactia of 21.8%, with cesarean section recipients showing a significantly higher risk (28.7%) compared with vaginal delivery (16.2%) [2]. This condition not only undermines the WHO’s global targets for exclusive breastfeeding (recommended for the first 6 months of life) but also exacerbates maternal postpartum depression and healthcare disparities [3].

In addition, with the development of modern society, the escalating work-related stress and the rising incidence of cesarean sections are key contributors to the growing prevalence of postpartum hypogalactia in women [3,4]. Currently, clinical management of postpartum hypogalactia primarily relies on hormone-based therapies designed to elevate prolactin levels and stimulate milk production [5]. Nevertheless, the administration of such prolactin-like agents carries inherent risks, including potential endocrine disturbances in nursing mothers and the possibility of drug residues accumulating in breast milk, which may lead to adverse health consequences for both the mother and the nursing infant [6,7,8]. These safety concerns underscore the urgent need to develop non-hormonal therapeutic alternatives for restoring lactation function.

Mammary epithelial cells (MECs) are the functional core of milk production [9,10,11,12]. Dysregulated secretion of hormones (e.g., prolactin) or impairment of mammary gland structural integrity compromises the milk-producing capacity of these cells [13,14,15]. Our previous research has demonstrated that 10 μM RepSox(R) can effectively induce fibroblasts to differentiate into chemically induced mammary epithelial cells (CiMECs) with functional capabilities for milk production. Furthermore, we have established that these CiMECs possess the potential to form mammary structures and produce milk in vivo [16]. This breakthrough provides a robust approach to obtain large quantities of MECs with regenerative and lactogenic functions, both in vitro and in vivo—addressing the limitations of standard primary MECs, which are scarce, difficult to isolate, and lose functional properties rapidly in culture.

Extracellular vesicles (EVs) have made remarkable progress in the study of tissue structure and function repair, especially in the use of EVs for disease treatment. Studies have shown that EVs can improve the animal model of fibrosis damage of various organs through various mechanisms. For example, the research on EVs secreted by Mesenchymal Stem Cells (MSCs) in dental and maxillofacial tissue regeneration has made rapid progress, showing the ability to promote the proliferation, differentiation, survival and migration of stem cells and precursor cells. In terms of nerve function repair after stroke, EVs that target the center and encapsulate specific molecules have shown potential to promote brain injury repair [17]. EVs have a huge advantage over traditional drug therapy, and their homing and targeting characteristics greatly avoid the defects that are easy to spread during drug therapy [18,19]. At the same time, the characteristics of easy transformation also make EVs valuable in the application process; they can add fluorescent labels for easy observation or add targets to improve the targeting, so that it has more sufficient imaging and operation space [20,21]. Moreover, because they are derived from the characteristics of the cell’s own secretion, their contents carry similar genetic information, proteins and lipids to the parent cells, which can be used as a test index for diagnosing diseases. This feature also avoids the risk of immune rejection, which is more conducive to clinical promotion than gene therapy [22]. Although the role of EVs in bone [23,24], fat [25], myocardial [26], nerve [27], and various oral tissue [28] regeneration has been extensively explored, innovative EV induction studies in chemically induced mammary epithelial cells (CiMECs) have not yet been investigated. Notably, CiMEC-derived EVs (CiMECs-EVs) are hypothesized to carry unique lactogenic and regenerative molecules—distinct from EVs derived from standard primary MECs (MECs-EVs). Unlike standard MECs, which are terminally differentiated and limited in functional plasticity, CiMECs are generated via transdifferentiation, a process that enriches their secretome with key regulators of mammary development and milk secretion. This makes CiMECs-EVs a more potent and targeted candidate for treating postpartum hypogalactia compared with MECs-EVs, which lack such induced functional enhancements.

Although EVs show great clinical potential, they face challenges in sustained-efficacy drug delivery, requiring repeated administration and risking dosage inaccuracy. GelMA, with its excellent biocompatibility, adjustable mechanical properties, photocuring properties, and good cell response, supports cell attachment, proliferation, and differentiation [29]. Notably, GelMA is liquid before injection and quickly forms a stable 3D structure post-injection, enabling sustained EVs release and long-term efficacy. Crucially, this property aids in restoring damaged breast tissue. Additionally, GelMA’s enzymatic degradability allows natural in vivo metabolism, avoiding the complexity and trauma of traditional implant surgeries and enhancing treatment safety. Thus, injectable GelMA encapsulating EVs presents a promising strategy for lactation-related diseases.

In this study, CiMECs-EVs were found to induce fibroblasts to transdifferentiate into milk-producing mammary epithelial cells, with the molecular mechanisms of this induction and lactation promotion explained at transcriptional and metabolic levels. Moreover, GelMA@CiMECs-EVs were proven to ameliorate lactation function in postpartum mice with lactation defects. In conclusion, this study innovatively presents a method of using CiMECs-EVs to modulate mammary epithelial cell fate and restore lactation, while GelMA application offers a new strategy for effective clinical treatment of insufficient postpartum lactation.

## 2. Method Details

### 2.1. Cell Culture

Goat ear fibroblasts (GEFs) were obtained from primary isolation in our laboratory. Ear margin skin biopsies were collected from a single healthy adult goat to ensure genetic consistency. Primary isolation was performed using the tissue block method. Briefly, the ear tissue samples were thoroughly sterilized with 75% ethanol, washed repeatedly with sterile PBS containing 2% penicillin–streptomycin, and then minced into approximately 1 mm^3^ pieces. The tissue pieces were seeded onto cell culture dishes and cultured in DMEM (Gibco, Grand Island, NY, USA, Cat.#11965-092) supplemented with 10% fetal bovine serum (WISENT, Saint-Jean-Baptiste, QC, Canada, Cat.#086-150) and 1% penicillin–streptomycin at 37 °C in a humidified atmosphere containing 5% CO_2_. Cells that migrated out from the explants were harvested, and the remaining tissue fragments were removed. To avoid phenotypic drift and cellular senescence, cells at passages 3 to 5 were used for all subsequent experiments. The purity of GEFs was confirmed by their typical spindle-shaped morphology, positive immunofluorescence for Vimentin (and negative for epithelial markers such as CDH1 and EPCAM), and further validated by qPCR. As shown in Supplementary Figure S1D, qPCR analysis demonstrated a significant downregulation of fibroblast-specific genes (*Col6a2*, *Fbn1*, *Vimentin*, *Fibin*) and an upregulation of epithelial marker genes (*Krt19*, *Itga6*, *Cdh1*, *Epcam*) during the induction process. Routine mycoplasma testing using a PCR-based detection kit confirmed that all cells used in this study were negative for mycoplasma contamination. Cells from a single goat were used for each independent replicate to minimize batch-to-batch variability.

### 2.2. Induction of MEC

GEF was uniformly inoculated into 60 mm cell culture dishes at a density of 5 × 10^5^, and when the cells were adherent to the wall and unfolded into a typical fibroblast morphology, they were replaced with 10 μM RepSox induction medium (the medium was changed every two days) and induced for eight consecutive days. Photographs were taken after each change in the medium, and the changes in the morphology of the induced cells were recorded.

Induction media formulations (100 mL example): Knockout DMEM/F12 (Life Technologies, Carlsbad, CA, USA, 11330-032), 40 mL; Neurobasal (Life Technologies, 21103-049), 40 mL; Knockout™ Serum Replacement (Gibco, 10828-028), 20 mL; 0.5% N2 (Invitrogen, Carlsbad, CA, USA, 17502-048); 1% B27 (Invitrogen, 17504044); glutamine (100×), 1 mL. The appropriate small molecule compounds were added at the above concentrations.

### 2.3. IF Staining

The IF staining procedure was performed as previously reported by our group [30]. After the cells were reached confluence, they were fixed with 4% paraformaldehyde (PFA) for 20 min, washed three times with blocking solution (PBS buffer with 100 mmol/L glycine and 0.3% BSA), blocked with 1% BSA for 1.5 h, and incubated with the corresponding primary antibodies at 4 °C overnight. The membranes were then incubated with secondary antibodies for 1.5 h at room temperature before being analyzed by imaging under a fluorescence microscope. For primary and secondary antibodies, see the Key resources table (Table 1).

### 2.4. qRT–PCR

The total RNA of the desired samples was extracted according to the instructions for TRIzol (Vazyme, Nanjing, China, Cat.#R401-01), the cDNA of the samples was subsequently obtained according to the Reverse Transcription Kit HiScript III RT SuperMix (Vazyme, Cat.#R323-01), and the cDNA of the samples was analyzed according to the Real-Time Fluorescence Quantitative PCR Kit ChamQ SYBR qPCR Master Mix (Vazyme, Cat.#Q711-02) steps.

The GenBank accession numbers for the corresponding goat genes analyzed by qPCR in this study are as follows: *GAPDH* (XM_005680968.3), *CDH1* (XM_005692180.3), *EPCAM* (XM_018055200.1), *KRT19* (XM_018065060.1), *ITGA6* (XM_018064673.1), *COL6A2* (XM_018052331.1), *FBN1* (XM_018054172.1), *VIMENTIN* (XM_018057155.1), and *FIBIN* (XM_005690019.3).

### 2.5. Oil Red O Staining

The cell samples were washed with PBS, and 10% neutral formaldehyde was added to fix the cells for 30 min to 1 h. During this period, the Oil Red O storage mixture was diluted to the working concentration with a ratio of red: DEPC water = 3:2, the mixture was filtered with filter paper, and the mixture was incubated at room temperature for 10 min. Subsequently, the mixture was added to the corresponding well plates for 10–30 min, and the specific amount added completely covered the bottom of the plate. Finally, the samples were rinsed with 75% alcohol to remove the background color and then observed under a microscope and imaged.

### 2.6. Western Blot

The cells were digested with trypsin (Gibco, Grand Island, NY, USA, Cat.#25200072) at the time of detection, washed by repeated centrifugation with PBS 3 times, the PBS was discarded, and the mixture was weighed. Cell lysate (RIPA) containing 10% protease inhibitor (PMSF) was added, and the mixture was lysed for 20 min on ice and then centrifuged at 1200 rpm at 4 °C for 10 min. The protein concentration was determined via an enzyme marker following the steps of the BCA kit (Beyotime, Shanghai, China, Cat.#P0012).

After the separation gel and concentration gel were prepared, the gel plate was placed in the electrophoresis tank, and the samples were sequentially loaded to start electrophoresis. After the proteins were transferred to the membrane, 5% skim milk powder was added, and the membrane was incubated at room temperature for 1 h (or overnight at 4 °C). The primary antibody was diluted with TBST according to the concentration recommended in the antibody instruction manual and incubated at room temperature for 2 h. After washing with TBST, the secondary antibody was diluted according to the concentration recommended in the antibody instruction manual and incubated on a shaking bed at room temperature for 40 min. After washing with TBST, the plate was put into the imager, and the color-developing solution was added to take pictures for recording.

### 2.7. Extracellular Vesicles Isolation Detection and Staining

After the cell culture medium was centrifuged at 3000× *g* for 10 min, the supernatant was transferred to a new centrifuge tube and placed on ice. In accordance with the instructions of the Extracellular Vesicles (EVs) Separation Kit (YeaSen Biotech, Shanghai, China, Cat.#41205ES10), the supernatant was mixed with EVs Extraction Reagent by vortexing, oscillated for 1 min, and then incubated at 4 °C for 2 h. The above mixture was transferred into EVs Purification Column (EP column) tubes, and the supernatant was extracted at 4 °C at 3000× *g* for 10 min. The mixture was centrifuged, and the liquid at the bottom of the tube was collected to isolate the EVs.

EVs were detected via nanoparticle tracking analysis (NTA). The EVs were diluted to 1 × 10^7^ particles/mL, and 2 mL of the EVs mixture at this concentration was prepared. The machine was switched on to clean the instrument lines, and no particles were observed in the field of view. The prepared EVs mixture was mixed with a 1 mL syringe, the particles in the field of view were observed, and the detection parameters, such as the number of measurements, the measurement time, and the dilution factor, were adjusted. After completing the test, the pipeline was rinsed, and the cuvette was cleaned.

EVs transmission electron microscopy (TEM) (Hitachi, Tokyo, Japan) detection: EVs were fixed via glutaraldehyde fixative to maintain their morphology and structure, and excess fixative was removed via buffer. The EVs were dehydrated stepwise using incremental concentrations of ethanol solution and impregnated with resin. After being embedded and cured on an ultrathin sectioning machine, the sections were cut into 60 nm ultrathin sections, which were collected on a copper mesh and photographed for documentation via a transmission electron microscope.

EVs PKH26 staining: EVs were subjected to a BCA protein concentration assay to determine the amount of protein. Purified EVs were mixed with PKH26 working solution and left to stand for 10 min according to the PKH26 reagent instructions (Sigma-Aldrich, St. Louis, MO, USA, Cat.#PKH26GL). Subsequently, 5–10 mL of PBS was added, mixed, and centrifuged at 100,000× *g* for 1.5 h to remove excess stain, and the bottom precipitate was resuspended in an appropriate amount of PBS. The particle-to-protein ratio was determined by correlating the total particle concentration measured by NTA with the total protein content quantified by the BCA assay. Based on this calculation, the EV dose used for in vivo administration (100 µg of protein) approximately corresponds to 1.5 × 10^10^ particles.

The isolation, characterization, and dose reporting of EVs were performed in accordance with the MISEV2023 consensus guidelines proposed by Welsh et al. [31].

### 2.8. MiRNA-Seq Library Construction and Sequencing

EVs were collected from RepSox-induced mammary epithelial cells on days 0, 4, and 8, and 2 biological replicates were set up in each group to construct a total of 6 small RNA sequencing libraries. The libraries were constructed according to the instructions of the GenSeq^®^ Small RNA Library Prep Kit (Wuhan, China), and then, the miRNA libraries were sorted via 6% nondenaturing polyacrylamide gel electrophoresis (PAGE). After passing the quality check, the libraries were subjected to Illumina HiSeq high-throughput sequencing.

### 2.9. MiRNA-Seq Data Processing and Analysis

The raw data were detected via FastQC (v0.11.9) software to generate HTML data reports, and the ineligible reads in the raw data were filtered and removed via Cutadapt (v4.4) software. The obtained clean reads were compared with the genomes of the studied species (goat; version number GCA_001704415.1) via Bowtie2 (v2.4.6) software, and the number of entries of the clean reads as well as the matching rate with the attended genome were calculated in exact match mode.

The data were processed for dimensionality reduction analysis via PCA and t-distributed stochastic neighbor embedding (tSNE) to verify sample consistency. Differential miRNAs of each sample were obtained via LIMMA (R-package limma v3.46.0) with a *p* value < 0.05 and |log2(fold_change)| ≥ 1.5 as the differential screening conditions. miRNAs were identified via TargetScan (https://www.targetscan.org/vert_71/) and MiRDB (https://mirdb.org/mirdb/index.html) for target mRNA prediction. Finally, the target mRNA set was analyzed for GO and KEGG enrichment.

### 2.10. MRNA-Seq Library Construction and Sequencing

Cell samples were collected from RepSox-induced mammary epithelial cells on days 0, 4, and 8, and mRNA was enriched with total RNA via oligo (dT) magnetic beads that bind to the poly(A) tail of the mRNA, removing the other types of RNA. The purified mRNAs were randomly disrupted by the addition of fragmentation buffer. A random hexamer primer, reverse transcriptase, was added to synthesize the first strand of cDNA using the fragmented mRNA as a template. Subsequently, buffer, dNTPs, RNase H and DNA polymerase I were added to the above reaction system to synthesize the second cDNA strand. The template mRNA was deleted, and double-stranded cDNA was synthesized. A dA tail (dA-tailing) was added to the 3′ end of the double-stranded cDNA, and purification and fragment size selection were performed via AMPure XP beads. The cDNA library was enriched via PCR amplification and constructed, and the library was sequenced on the machine after quality control.

### 2.11. MRNA-Seq Data Processing and Analysis

The steps of the processing and analysis methods were broadly similar to those of miRNA-seq.

### 2.12. Combined Analysis of miRNA-Seq and mRNA-Seq Data

The target mRNAs of the differential miRNAs identified via miRNA-seq were intersected with the differential mRNAs identified via RNA-seq by taking the intersection through the Venn diagram, and the miRNA–mRNA regulatory network was constructed via the STRING database and Cytoscape software (version 3.9.1).

### 2.13. Metabolomic Sequencing and Analysis

The samples were separated via an Agilent 1290 Infinity Ultra-High-Performance Liquid Chromatography (UHPLC) system (Agilent Technologies, Santa Clara, CA, USA) equipped with a HILIC column; the temperature, flow rate and injection volume were set for gradient elution; and then the samples were subjected to primary and secondary mapping.

After the samples were analyzed by chromatography and mass spectrometry as described above, the raw LC–MS data were obtained, and after the application of the metabolomics analysis software XCMS, the preliminary data matrix (elements: retention time, peak intensity, and mass–charge ratio) was obtained by baseline filtering → peak identification → integration → retention time correction → peak alignment. The preliminary data matrix (elements: retention time, peak intensity, and mass–charge ratio) was obtained by removing missing values and then retaining the variables that had a nonzero value of 80% or more in at least one group of samples to fill in the above vacancies. After the missing values were removed, at least one set of variables with more than 80% of the nonzero values in the sample was retained to fill in the vacant values. Normalized data matrix: variables with RSDs (relative standard deviations) less than 30% of the QC samples were retained and logarithmically processed (log10) to obtain the final data table. These data were analyzed in the HMDB (http://www.hmdb.ca/) and Metlin (https://metlin.scripps.edu/) databases to match the final metabolite information.

Data models were constructed via PCA, PLS-DA and OPLS-DA, and the models were evaluated for correction. Differentially abundant metabolites were screened via t tests (screening condition: *p* value < 0.05), fold changes (screening condition: fold change ≥ 1.5) and variable importance projection values (screening condition: VIP > 1) in the OPLS-DA model. The above differentially abundant metabolites were passed through the KEGG database (https://www.kegg.jp/kegg/pathway.html), the degree of their enrichment in the corresponding metabolic pathways was compared according to the frequency of enrichment of each differentially abundant metabolite in the database, and the significant metabolic pathways were identified according to the enrichment results.

### 2.14. Preparation of GelMA@CiMECs-EVs

Dissolve 0.05 g LAP (photoinitiator, final concentration 0.25% *w*/*v*) in 20 mL PBS in an amber bottle, mix thoroughly, and heat in a 45 °C water bath for 15 min with shaking every 5 min. Cool the mixture to 4 °C for later use. Mix the cooled solution with GelMA, heat at 65 °C in the dark for 25 min with periodic shaking every 5 min. Filter the resulting solution through a 0.22 μm filter, incubate at 37 °C in the dark, and then mix with PKH26-CiMECs-EVs at the desired concentration. Prior to in vivo administration, the mixture was photocrosslinked under UV light (wavelength: 365 nm, light intensity: 10 mW/cm^2^) for 30 s, at a fixed exposure distance of approximately 5 cm, to form a stable hydrogel network.

### 2.15. Background and Grouping of Experimental Mice

In this study, we used 60 female and 20 male KM mice of 7–8 weeks of age, all of which were sexually mature enough to conceive but had not yet given birth, and all of which were sexually mature enough to have experienced offspring.

After the estrous cycle of the females was examined as described above, the females in estrus were selected for mating at a ratio of female: male = 3:1. After that, the males and females were housed in separate cages. Female mice with similar body weights were used as experimental mice, and the number of litters per female was adjusted to 8 for the sake of litter survival rate and body weight gain; the litter weights (8 mice) should be kept as consistent as possible between groups. Random number table method was used to group the mice as listed in the Table 2 below.

To ensure robust statistical power for the physiological measurements, n = 6 individual female KM mice were allocated to each experimental group in this study.

### 2.16. Establishing Postnatal Lactation-Defective Model Mice

1. Preparation of Bromocriptine Solution

Bromocriptine tablets (Gedeon Richter, Budapest, Hungary; specification: 2.5 mg per tablet) were used to construct the postpartum lactation deficiency model in mice. The target dose of bromocriptine (molecular formula: C_32_H_40_BrN_5_O_5_) was set at 1.6 mg/kg body weight (BW) based on previous preclinical studies and adjusted for the metabolic characteristics of KM mice. For solution preparation,

- First, bromocriptine tablets were ground into a fine powder using a sterile mortar under light-protected conditions to ensure uniform dissolution.
- The powder was dissolved in sterile normal saline (0.9% NaCl) and gently stirred at 4 °C overnight; ultrasonic treatment (40 kHz, 10 min) was applied midway to accelerate dissolution and avoid precipitation.
- The final mother liquor was filtered through a 0.22 μm sterile filter membrane to remove undissolved particles, aliquoted into light-protected centrifuge tubes, and stored at 4 °C for no more than 7 days to maintain stability.
- For the blank control group (normal saline group), an equal volume of sterile normal saline was administered by gavage to eliminate confounding effects from experimental manipulation. Before each gavage, the bromocriptine solution was rewarmed at room temperature for 40 min and gently inverted 5–8 times to ensure homogeneity, avoiding temperature shock to the mice.

2. Modeling Protocol and Timing

- Female KM mice were monitored closely during parturition, and the day of complete delivery (all pups born) was recorded as day 0.
- To allow maternal adaptation to lactation and postpartum recovery, modeling was initiated on postpartum day 3. In our study, the therapeutic intervention was initiated concurrently with the bromocriptine induction on postpartum day 3. This parallel design was selected to ensure the survival and normal growth of the pups. Delaying the therapeutic intervention until a fully established lactation deficiency model was confirmed (approximately postpartum day 13) would inevitably lead to severe malnutrition and significant mortality of the litters, thereby compromising the feasibility and reliability of evaluating in vivo therapeutic efficacy.
- Gavage was performed at 9:00 AM daily for 10 consecutive days using a 1 mL sterile gavage needle. The administration volume was calculated as 10 μL/g BW (e.g., 200 μL for a 20 g mouse) to ensure accurate dose delivery. During gavage, the mouse was gently restrained with the head fixed in a neutral position to avoid esophageal injury, and the needle was inserted slowly ≤ 2 cm depth) to minimize stress.
- Throughout the modeling period, mice were housed under standardized conditions (temperature: 22 ± 2 °C, humidity: 55 ± 5%, 12 h light/dark cycle) with ad libitum access to food and water. Litter size was standardized to 8 pups per mother to ensure consistent lactation demand across groups, and any dead pups were replaced with age-matched foster pups within 24 h.

3. Criteria for Successful Model Establishment

The lactation deficiency model was considered successful when all of the following four indicators were met, verified on postpartum day 13 (after 10 days of modeling):

- Hourly lactation volume: The average hourly lactation volume (measured by the pup weight gain method, as described in “Collection and analysis of mouse indicators”) was ≤1.3 g, which was significantly lower than the normal lactation baseline (≥1.7 g/h) of KM mice.
- Mammary gland index: The mammary gland index (calculated as [mammary gland wet weight/mouse BW] × 100%) was <7%, reflecting structural impairment of mammary tissue.
- Histopathological features: Hematoxylin–eosin (HE) staining of mammary gland sections showed narrowed and shrunken mammary ducts with sparse luminal space, and minimal to no milk filling was observed in the ductal lumen, consistent with lactation dysfunction.
- Lactation-specific protein expression: Immunofluorescence (IF) staining revealed that the positive rate of PRLR (a key lactation-specific protein) in mammary tissue was ≤15% of that in the normal pregnancy and delivery group (regular group), confirming defective milk synthesis function.

### 2.17. GelMA@CiMECs-EVs Treatment Model Mice

The female mice in the corresponding experimental groups were modeled with bromocriptine by gavage at 9:00 and treated with injections of GelMA@CiMECs-EVs at 17:00 each day. The total cumulative dose of CiMECs-EVs administered to each mouse over the 10-day therapeutic period was 1000 µg (100 µg per injection, once daily).

The injection steps were as follows:

1. For each female mouse in the injection group, we ensured that the skin around the inguinal papillae (fourth and fifth pairs) was fully exposed.
2. Fixing the female mice: unlike the upright position of the gavaged female mice, the female mice were placed on their backs to expose the inguinal papillae for subsequent injection.
3. Using straight forceps, the skin around the fourth pair of nipples was pinched up, an insulin needle aspirating the corresponding concentration of GelMA@CiMECs-EVs was inserted under the nipple, and the drug was pushed slowly.
4. The sham-operated group used the same injection method, and at the same time, aspirated the same volume of PBS as GelMA@CiMECs-EVs and injected it into the same location of the mammary gland to ensure a good control with the experimental group.

### 2.18. Collection and Analysis of Mouse Indicators

1. Body weight loss of female mouse: On the 3rd day after delivery, i.e., the 1st day of the start of modeling (and the start of treatment), the body weight of female mouse was the initial body weight. On the last day of modeling (also the last day of treatment), the body weight of the female mouse was the final body weight. The reduction in the body weight of the female mouse = final body weight − initial body weight.
2. Net litter weight gain: The litter weight of the female mice (8 pups) on the 3rd day after delivery, i.e., the 1st day after the start of modeling (and the start of treatment), was the initial litter weight. On the last day of modeling (also the last day of treatment), the litter weight of the pups (8 pups) was the final litter weight. Net litter weight gain = final litter weight − initial litter weight.
3. Hourly lactation of female mice: During the period when the female mice were feeding their litters, to ensure that the amount of milk produced by the female mice was sufficient for the normal development and growth of each group of littermates, it was not convenient to accurately measure the amount of milk produced by the female mice in a single hour. By fixing the number of pups in each litter (8 pups) and the initial weight of the mothers, the hourly lactation rate of the female mice could be calculated via the following formula. W1: litter weight (8 pups) weighed at 9:00 a.m.; W2: litter weight (8 pups) of the mother and pups after they have been separated from each other for 4 h; W3: litter weight (8 pups) of the mother and pups after they have been fed together in a cage for 1 h. Formula: Hourly lactation time of female mouse = W3 − W2 + (W1 − W2)/4.
4. The tissue organ index was calculated as follows: tissue organ index = organ (tissue) weight/body weight.

### 2.19. HE Staining

The organs of each group of mice were fixed, washed, dehydrated, transparent, embedded in dipping wax, sectioned and deparaffinized for staining, and then dried and sealed in an ultra-clean bench and photographed and recorded under a microscope.

### 2.20. Frozen Section and Immunofluorescence Staining of Tissues

After the samples were taken, the samples were placed in a quick-freezing box with OCT embedding agent added and quick-frozen in liquid nitrogen, and then placed in a frozen sectioning machine for slicing. The samples were rewarmed at room temperature for 20 min and soaked in PBS for 10 min to remove the OCT embedding agent. The sections were incubated with 0.5% TritonX-100 solution at room temperature for 30 min. Non-specific sites were closed with PBS-Tween solution, incubated according to the antibody instructions, and sealed with drops of anti-fluorescence quencher.

### 2.21. Intracellular miRNA Transfection

Transfection was performed using Lipofectamine 2000 (Invitrogen, Carlsbad, CA, USA) transfection reagent. Mimics and inhibitors of key miRNAs were synthesized by Genepharma, with their sequences listed in Tables S1 and S2. Three biological replicates were set up for each group.

One day prior to transfection, fibroblasts were seeded into 6-well plates at a density of 3 × 10^5^ cells per well to ensure a cell confluency of 60–70% at the time of transfection. For each well, 2 μL of Lipofectamine 2000 transfection reagent was mixed with 50 μL of Opti-MEM medium, gently vortexed, and incubated at room temperature for 5 min. In a separate step, miRNA mimics or inhibitors (final concentration of 100 nM, with equal concentrations of each miRNA in each group) were mixed with 50 μL of Opti-MEM medium. The two mixtures were then combined and incubated at room temperature for 20 min to form Lipofectamine 2000–miRNA complexes. The original medium in the 6-well plates was discarded and replaced with 1 mL of serum-free and antibiotic-free Opti-MEM medium, followed by the dropwise addition of the transfection complexes. The plates were gently rocked to ensure uniform mixing, and then incubated at 37 °C with 5% CO_2_ for 6 h. After incubation, the transfection medium was removed and replaced with complete medium containing 10% fetal bovine serum (FBS) and 1% penicillin–streptomycin. Transfection was performed every two days, and subsequent sample collection was carried out according to the experimental protocol.

### 2.22. Detection of miRNA Expression Levels and Markers via qPCR

Total RNA was extracted from the cells using TRIzol reagent following the manufacturer’s instructions. After quantification and quality assessment of the RNA, cDNA was synthesized using the stem-loop method with the Mir-X miRNA First—Strand Synthesis Kit (Takara Bio, Kusatsu, Shiga, Japan, Cat.#638313). Specific primers for the target miRNAs and the internal control U6 snRNA were designed and are detailed in Table S3. qPCR was performed using TB Green Premix Ex Taq II (Takara, RR820A). Each sample was analyzed in triplicate, and the relative expression levels were calculated using the 2^−ΔΔCt^ method. The procedure for detecting markers was identical to that described above.

### 2.23. Luciferase Reporter Assay

The 3′UTR fragments of target genes (*TGFβR1*, *EGFR*, and *PRLR*) containing the predicted miRNA binding sites were cloned into the pmirGLO dual-luciferase vector (Promega, Madison, WI, USA). Mutant 3′UTR vectors with mutations in the miRNA seed sequence binding sites were also constructed. HEK293T cells were seeded into 96-well plates and co-transfected with 50 ng of the reporter plasmids and 50 nM of miRNA mimics or negative control using Lipofectamine 2000 (Invitrogen) according to the manufacturer’s instructions. After 48 h of incubation, firefly and Renilla luciferase activities were measured using the Dual-Luciferase Reporter Assay System (Promega) following the manufacturer’s protocol. Renilla luciferase activity was used as an internal control, and the relative luciferase activity of each group was normalized to the negative control group.

## 3. Results

### 3.1. CiMECs-EVs Can Induce Fibroblasts into Mammary Epithelial Cells

**Figure 1:**
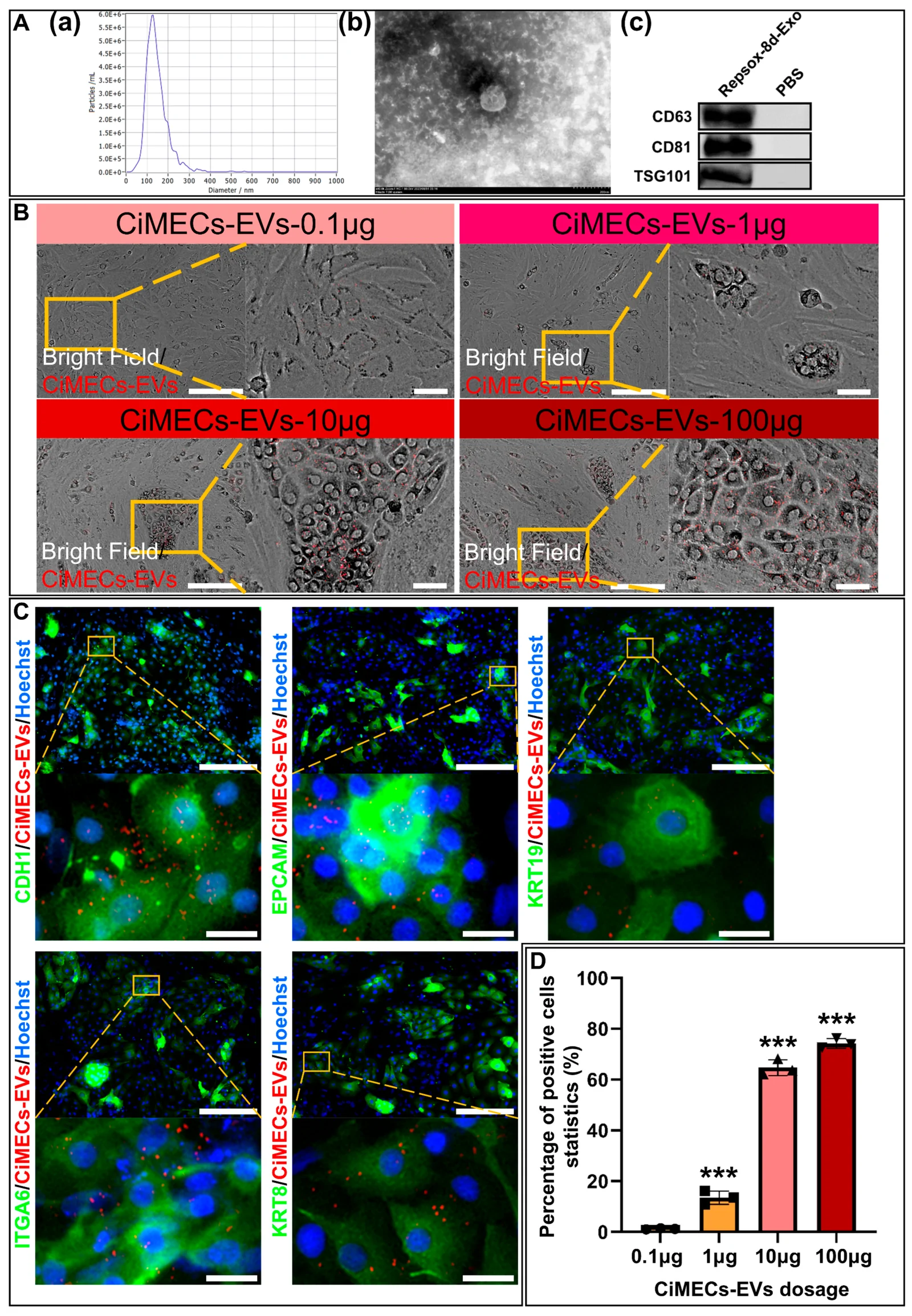
CiMECs-EVs have the ability to induce a mammary epithelial-like phenotype in fibroblasts. (**A**) (**a**) CiMECs-EVs particle size detection; (**b**) CiMECs-EVs transmission electron microscopy (TEM) detection, scale = 200 nm; (**c**) the expression of CiMECs-EVs, marker proteins CD63 (26 kDa), CD81 (30 kDa), TSG101 (44 kDa) in RepSox-8d-EVs and PBS was detected by Western blot. (**B**) PKH26-CiMECs-EVs 0.1 μg, 1 μg, 10 μg, 100 μg (red fluorescence)-induced fibroblasts. The concentrations of 10 μg and 100 μg induced a typical mammary epithelial cell morphology. (Scale bars, 200 μm. Magnification scale bars, 40 μm.) (**C**) PKH26-CiMECs-EVs 10 μg-induced mammary epithelial cell marker proteins CDH1, EPCAM, KRT19, ITGA6, KRT8 (green fluorescence) immunofluorescence staining was positive. PKH26-CiMECs-EVs (red fluorescence) were uniformly distributed around the inducing mammary epithelial cells. (Scale bars, 200 μm. Magnification scale bars, 20 μm.) (**D**) The bar chart shows the percentage of breast epithelial cells (positive cells) under different doses of CiMECs-EVs (0.1 μg, 1 μg, 10 μg, 100 μg). The positive rate gradually increases as the dose of CiMECs-EVs increases. (*** *p* < 0.001, one-way ANOVA).

In accordance with the methodology previously reported by our research group [16], RepSox was employed to induce fibroblasts to transdifferentiate into mammary epithelial cells (CiMECs) (Figure S1), and this process was validated through RNA-Seq analysis (Figures S2–S4). The supernatants collected during the induction were subjected to a commercial EV isolation kit (YeaSen Biotechnology) to isolate EVs, referred to as CiMECs-EVs. The size and morphology of CiMECs-EVs were assessed using particle size analysis and transmission electron microscopy, while Western blotting confirmed the presence of marker proteins CD63, CD81, and TSG101, which tested positive in comparison with the PBS control group (Figure 1A). This indicates that the isolated CiMECs-EVs exhibited normal characteristics in terms of size, morphology, and protein markers. To facilitate subsequent experiments, CiMECs-EVs were labeled with PKH26. To evaluate the inductive capacity of PKH26-labeled CiMECs-EVs, fibroblasts were treated with varying concentrations of CiMECs-EVs (0.1 μg, 1 μg, 10 μg, 100 μg), revealing that CiMECs-EVs at concentrations between 10 μg and 100 μg significantly induced a mammary epithelial-like phenotype in fibroblasts (Figure 1B). Immunofluorescence detection demonstrated that CiMECs-EVs induced the expression of specific markers associated with mammary epithelial cells (CDH1, EPCAM, KRT19, ITGA6, KRT8), while red fluorescence from PKH26-labeled CiMECs-EVs was observed within the induced mammary epithelial cells (Figure 1C). With increasing dosage of CiMECs-EVs, the rate of mammary epithelial cells exhibited a gradual increase; however, the discrepancy in rates became less pronounced between 10 μg and 100 μg, thereby offering a dosage reference for subsequent in vivo validation (Figure 1D). Notably, the induced cells at concentrations of 10 μg and 100 μg displayed a distinctly granular cytoplasmic appearance (Figure 1B and Figure S1B). This morphological characteristic is highly indicative of a typical glandular/secretory phenotype. Importantly, the accumulation of intracellular lipid droplets in these cells was further confirmed by positive Oil Red O staining (Figure S1E), providing direct evidence linking the granular morphology to functional milk-fat synthesis. Therefore, these morphological observations validate the successful transition from a fibroblast into a functional glandular fate. Thus, it can be concluded that CiMEC-EVs possess a robust ability to induce fibroblasts to acquire a mammary epithelial-like phenotype.

### 3.2. Regulation of CiMECs-EVs–Derived miRNA-Induced Fibroblast Transdifferentiation into Mammary Epithelial Cells

**Figure 2:**
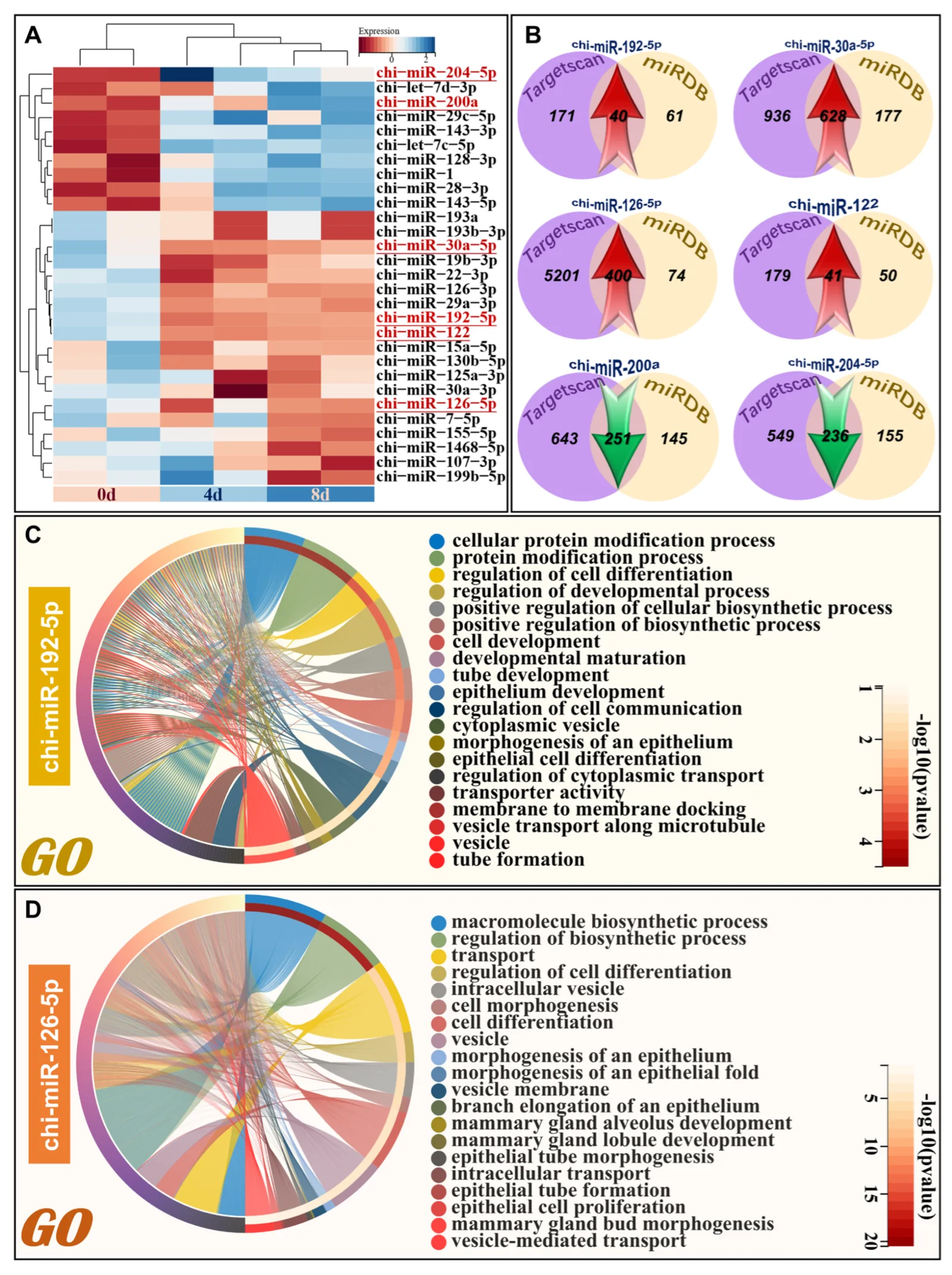
Regulation of CiMECs-EVs–derived miRNA induces fibroblast transdifferentiation into mammary epithelial cells. (**A**) Differential heat maps show the differential miRNAs of CiMECs-EVs-0d, -4d, -8d. Among them, chi-miR-192-5p, chi-miR-126-5p, chi-miR-30a-5p, chi-miR-122, chi-miR-200a, and chi-miR-204-5p had significant differences. (**B**) TargetScan and miRDB websites predict the intersection of target genes of key differential miRNAs. (**C**,**D**) GO enrichment analysis of target genes of key differential miRNAs.

**Figure 3:**
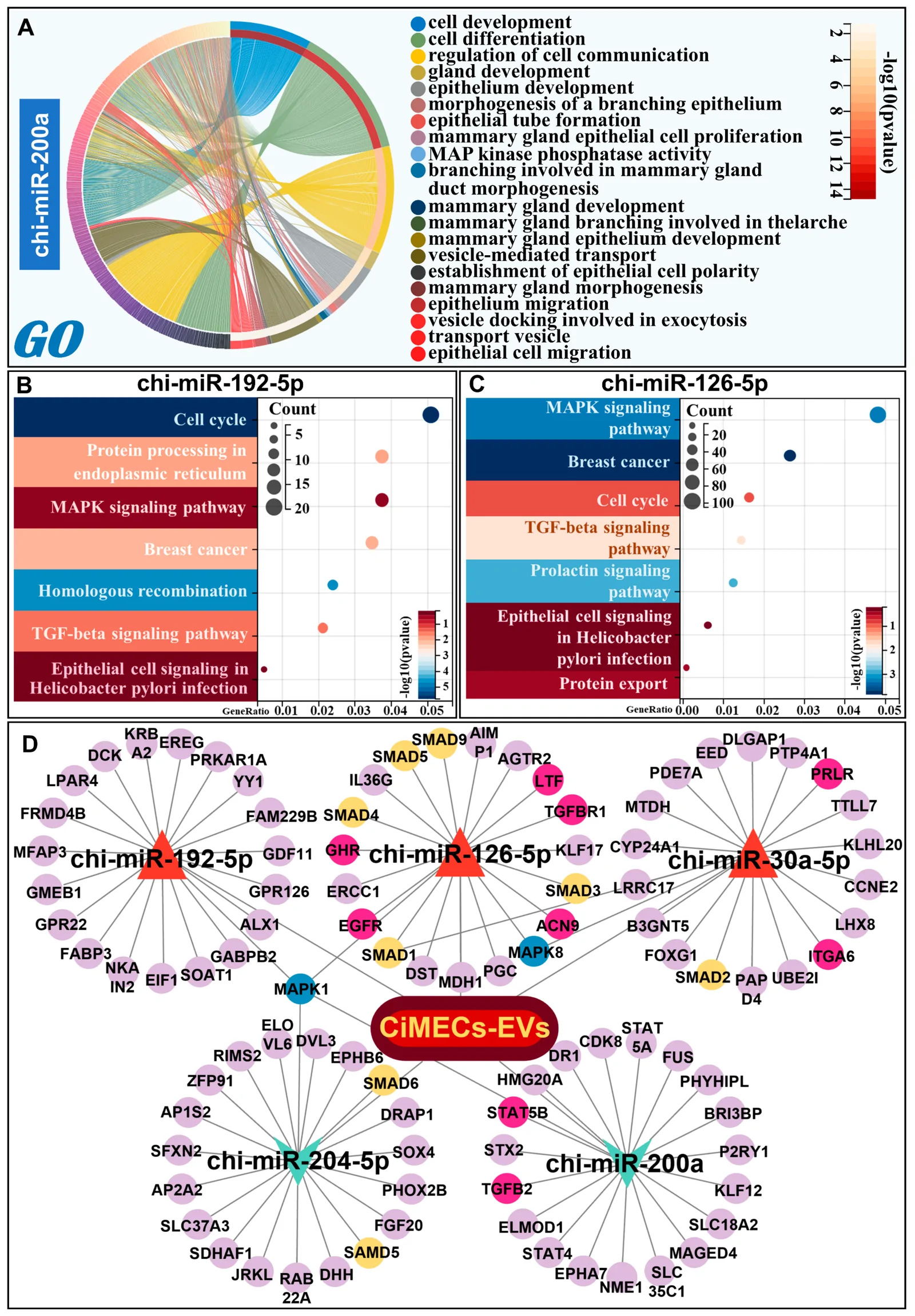
GO and KEGG enrichment analyses of key miRNAs and the network diagram of CiMECs-EVs and key miRNAs. (**A**) GO enrichment analysis of target genes of key differential miRNAs. (**B**,**C**) KEGG analysis of the target gene of key differential miRNAs. (**D**) Joint analysis of miRNA-Seq of CiMECs-EVs and RNA-Seq of CiMECs. In subgraph (**D**), red triangles and red nodes indicate up-regulated miRNAs, whereas green triangles and blue nodes indicate down-regulated miRNAs.

Next, CiMECs-EVs-0d, -4d, and -8d were collected for miRNA sequencing analysis. Through pairwise comparison of the above three groups, different miRNA screening was conducted according to the criteria of |Fold change| ≥ 1.5, P ≤ 0.05. The results showed that chi-miR-192-5p, chi-miR-126-5p, chi-miR-30a-5p, chi-miR-122, chi-miR-200a, and chi-miR-204-5p had significant differences (Figure 2A; Figure S5) during the transdifferentiation process. Target genes of the above six different miRNAs were predicted using TargetScan and the miRDB website, and the intersection of the two miRNAs was used to determine the target gene set (Figure 2B). In order to clarify the specific functions of the above six different miRNAs, GO and KEGG enrichment analyses were performed. The results showed that chi-miR-192-5p was mainly related to cell development, cell differentiation and cytoplasmic vesicles. The other five differential miRNAs were mainly related to mammary gland bud morphogenesis, mammary gland development, and miRNA binding (Figure 2C,D, Figure 3A–C, Figure S6 and Figure S7).

The results of miRNA-Seq of CiMECs-EVs combined with those of RNA-Seq of CiMECs showed that chi-miR-126-5p, chi-miR-30a-5p, and chi-miR-200a can target the mammary epithelial cell marker genes *LTF*, *GHR*, *ACN9*, *EGFR*, *PRLR*, *ITGA6*, and *STAT5B*. At the same time, chi-miR-126-5p can also target *TGFβR1* and *SMAD3*, which regulate the CiMECs generation discovered by our research group previously [32] (Figure 3D). The above analysis suggests that miRNAs contained in CiMEC-EVs may have the ability to induce fibroblasts to reprogram into mammary epithelial cells.

To validate the key miRNAs identified by miRNA-seq of the CiMECs-EVs mentioned above, qPCR analysis revealed that in CiMECs-EVs-8d vs. -0d, chi-miR-192-5p, chi-miR-126-5p, and chi-miR-30a-5p were upregulated, while chi-miR-204-5p and chi-miR-200a were downregulated, which is consistent with the miRNA-seq data (Figure S8A). To further validate the direct molecular interactions, dual-luciferase reporter assays confirmed that chi-miR-126-5p directly targets the 3′UTRs of *TGFβR1* and *EGFR*, while chi-miR-30a-5p directly targets *PRLR* (Figure S11).

To further confirm the role of these key miRNAs, we transfected inhibitors of chi-miR-192-5p, chi-miR-126-5p, and chi-miR-30a-5p, as well as mimics of chi-miR-204-5p and chi-miR-200a, into fibroblasts. Subsequent induction with CiMECs-EVs using the same method, followed by immunofluorescence (IF) staining and statistical analysis, showed that the induction efficiency was significantly reduced. This indicates that these key miRNAs play a crucial role in the fate determination of mammary epithelial cells (Figure S8B).

### 3.3. CiMECs-EVs–Derived Metabolites Demonstrated That Fibroblasts Were Induced to Transdifferentiate into Mammary Epithelial Cells

**Figure 4:**
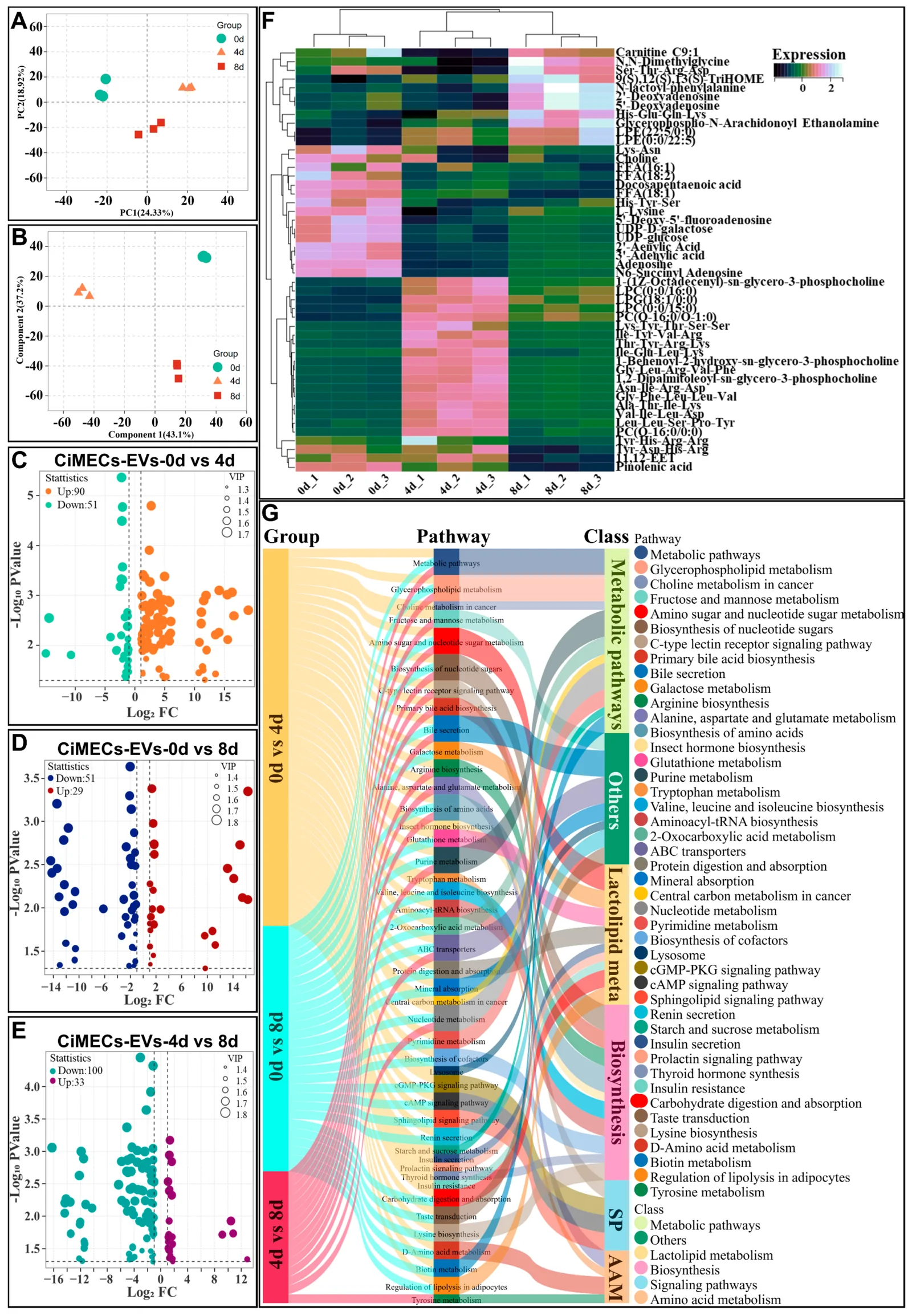
Metabolome analysis revealed that CiMECs-EVs can regulate and induce the fate and function of mammary epithelial cells. (**A**) CiMECs-EVs-0d, -4d, -8d metabolome PCA score chart; (**B**) PLS-DA scores of CiMECs-EVs-0d, -4d, -8d metabolome; (**C**–**E**) CiMECs-EVs-0d, -4d, -8d differential metabolite volcano maps. (**F**) CiMECs-EVs-0d, -4d, -8d differential metabolite clustering heat map. (**G**) KEGG enrichment analysis of CiMECs-EVs-0d, -4d, -8d differential metabolites.

In order to further reveal the specific metabolite composition of CiMECs-EVs, metabolomic analysis of CiMECs-EVs-0d, -4d and -8d was performed. PCA analysis was used to cluster the above samples for dimensionality reduction. PLS-DA established the relationship model between metabolite expression and sample category and realized the prediction of sample category and the screening of auxiliary marker metabolites (VIP > 1). Based on PLS-DA, OPLS-DA was used for correction, and noise irrelevant to classification information was filtered to improve the analytical ability and effectiveness of the model (Figure 4A,B and Figure S8C). Differential metabolites were screened using the criteria of |Fold change| ≥ 2, P ≤ 0.05 (Figure 4D–F). Through KEGG enrichment analysis of these differential metabolites, it was found that they were mainly related to the prolactin signaling pathway, galactose metabolism, glutathione metabolism, tyrosine metabolism, etc., which are related to milk fat metabolism and amino acid metabolism. Biosynthesis and other aspects are closely related (Figure 4G). According to the functional analysis of the above metabolome, CiMECs-EVs may generate related metabolites that promote the development of mammary cells and improve the function of lactation through milk fat metabolism and biosynthesis to maintain the homeostasis of mammary epithelial cells after induction.

### 3.4. GelMA@CiMECs-EVs Have Excellent Properties for Therapeutic Applications

**Figure 5:**
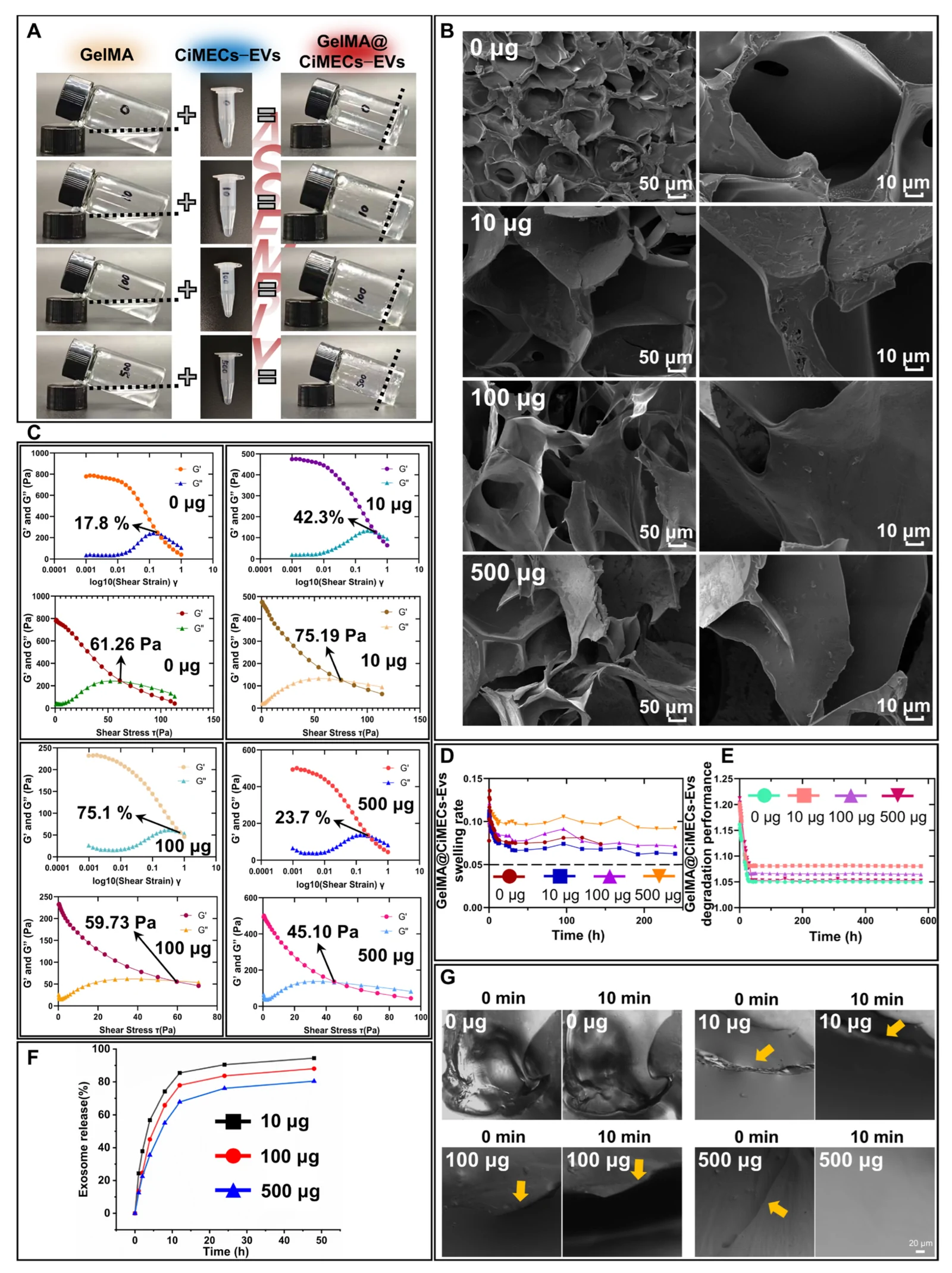
Characterization and performance test of GelMA@CiMECs-EVs. (**A**) Representative images of the GelMA, CiMECs-EVs and GelMA@CiMECs-EVs at different concentrations. The dotted lines in panel (**A**) mark the liquid-level position of hydrogel samples. (**B**) SEM images of GelMA, CiMECs-EVs and GelMA@CiMECs-EVs at different concentrations. (**C**) The strain-dependent oscillatory shear rheological properties of GelMA@CiMECs-EVs at different concentrations. (**D**,**E**) Swelling rate and degradation performance of GelMA@CiMECs-EVs at different concentrations. (**F**) Release rate of GelMA@CiMECs-EVs. (**G**) GelMA@CiMECs-EVs have good self-healing ability. Yellow arrows indicate the cutting sites for evaluating the self-healing performance of hydrogels.

To optimize the self-assembly of GelMA and CiMECs-EVs into a gel with enhanced swelling, degradation, and drug release properties, we set up CiMECs-EVs concentration gradients at 0 μg, 10 μg, 100 μg, and 500 μg, combined with 5% GelMA. Results showed that all GelMA@CiMECs-EVs concentrations gelled under UV light, turning semi-solid in 15 s and fully gelled in 30 s (Figure 5A). SEM revealed a porous, mesh-like structure with CiMECs-EVs on the surface across all groups (Figure 5B). Rheology showed that at shear strains exceeding 17.8%, 42.3%, 75.1%, and 23.7% for the 0 μg, 10 μg, 100 μg, and 500 μg groups, respectively, G″ surpassed G′, indicating a shift from elastic to viscous behavior. Viscosity increased from 0 μg to 100 μg, and then dropped at 500 μg (Figure 5C).

Notably, the unexpected shifts observed at the 500 μg group, where viscosity decreased and degradation slowed compared with the 100 μg group, can be comprehensively explained by physicochemical interference. First, the high density of EVs physically occupies the interstitial spaces of the GelMA polymer chains, acting as a steric hindrance that obstructs the free radical polymerization process, thereby leading to a less dense crosslinking network. Second, the highly turbid dispersion of 500 μg EVs scatters the incident 365 nm UV light, which reduces the excitation efficiency of the LAP photoinitiator and further decreases the effective crosslinking density within the hydrogel matrix. Finally, the abundant protein and lipid cargoes of the concentrated EVs compete for water binding sites within the hydrophilic GelMA network, altering the hydration state of the hydrogel and consequently affecting its viscosity and degradation profile. These mechanistic explanations align well with the macroscopic behaviors observed in Figure 5C–E, where the 500 μg group exhibited a sparser viscoelastic network and slower hydration/degradation dynamics.

Swelling rate and degradation rate are critical for assessing drug loading, release, and in vivo degradability. The 500 μg and 100 μg groups showed superior EV loading, while the 100 μg group degraded better than the 500 μg group. Although the 10 μg group degraded the fastest, its loading capacity was lower (Figure 5D,E). For drug release, all groups released over 30% of EVs in 5 h and over 70% in 24 h, indicating effective EV release (Figure 5F). Self-healing tests showed all groups repaired quickly within 10 min, preventing EV burst release (Figure 5G). Overall, GelMA@CiMECs-EVs gels demonstrated excellent injectability, high loading capacity, degradability, and self-healing ability.

Based on the above cellular experiments and material characterization results, it can be concluded that 100 μg of GelMA@CiMECs-EVs achieves an excellent therapeutic effect while reducing the dosage of EVs. Therefore, the concentration of 100 μg was adopted in subsequent animal experiments for the in vivo verification of therapeutic efficacy.

### 3.5. GelMA@CiMECs-EVs Have the Ability to Restore Lactation in Mice with Lactation Deficiency

**Figure 6:**
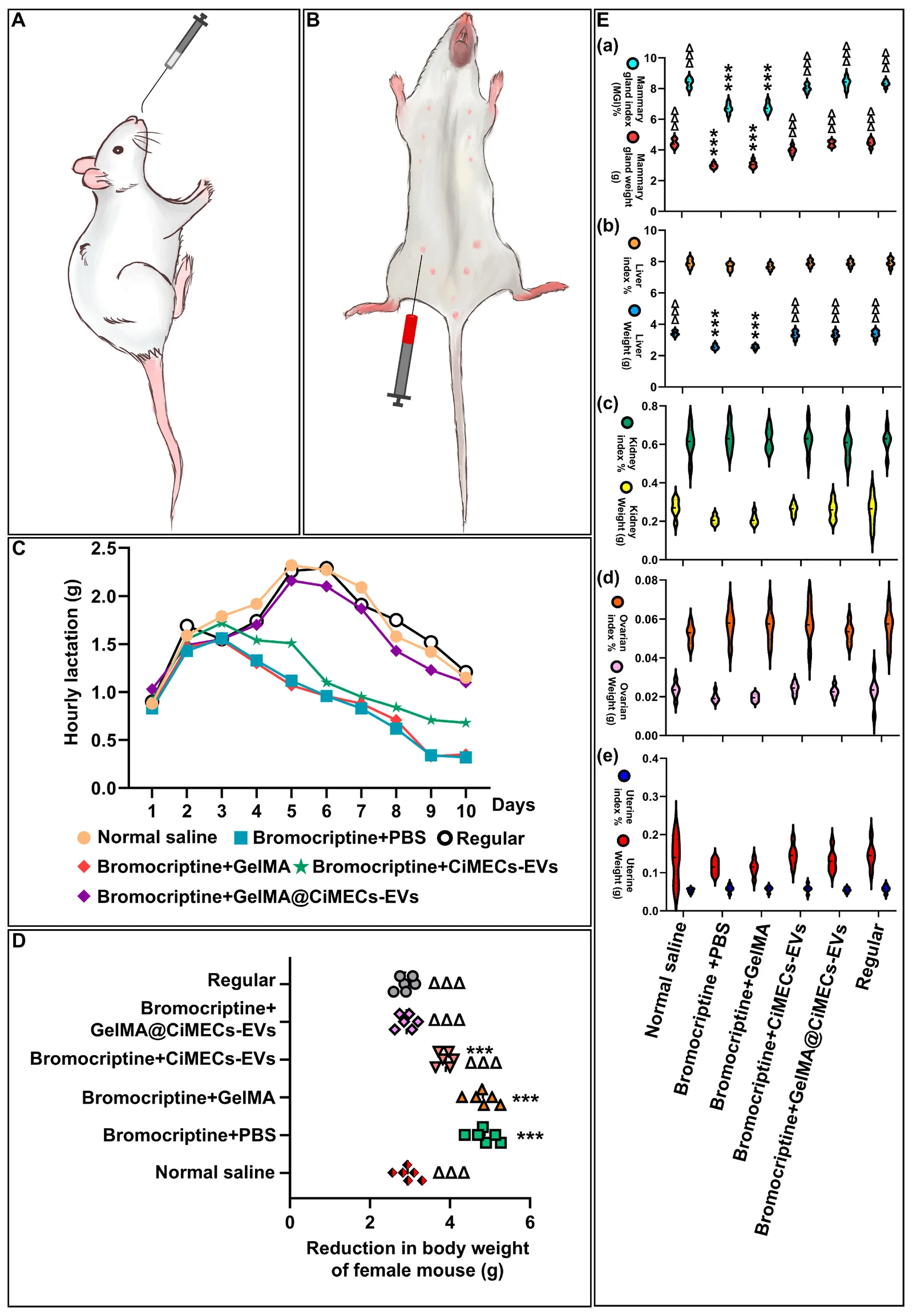
GelMA@CiMECs-EVs demonstrate the capability to restore lactation in mice with lactation deficiency. (**A**) Schematic diagram of gavage of lactation-deficient model. (**B**) GelMA@PKH26-CiMECs-EVs treatment diagram. (**C**) The statistical chart illustrating the variations in hourly milk production across the six experimental groups. (**D**) Statistical chart of body weight change in female mice in each group. (**E**) Violin plots showing the statistical analysis of (**a**) mammary gland index, (**b**) liver index, (**c**) kidney index, (**d**) ovary index, and (**e**) uterus index across all groups. (* represents significance analysis compared with the normal saline group; Δ represents a significant analysis compared with the bromocriptine + PBS group; mean ± SEM, *n* = 6 individual mice per group, *** *p* < 0.001, ΔΔΔ *p* < 0.001, one-way ANOVA.)

To evaluate the therapeutic potential of GelMA@CiMECs-EVs, the composite was administered to bromocriptine-induced lactation-deficient mice (Figure 6A,B). Compared with the model group (bromocriptine + PBS), intramammary injection of 100 μg GelMA@PKH26-CiMECs-EVs achieved significant improvements in hourly lactation volume and alleviation of maternal weight loss, with therapeutic effects comparable to the normal saline and regular groups (Figure 6C,D). Notably, the GelMA-only group (bromocriptine + GelMA) exhibited a lactation curve and body weight changes nearly identical to the model group, demonstrating that the hydrogel itself provides no lactogenic benefit. Additionally, the free CiMECs-EVs group (bromocriptine + CiMECs-EVs) showed moderate therapeutic effects during the first three days; however, its lactation yield decreased sharply after day 4, ultimately remaining significantly inferior to the GelMA@CiMECs-EVs group. These findings underscore the essential role of GelMA hydrogel encapsulation in ensuring sustained EV release and optimal in vivo efficacy.

**Figure 7:**
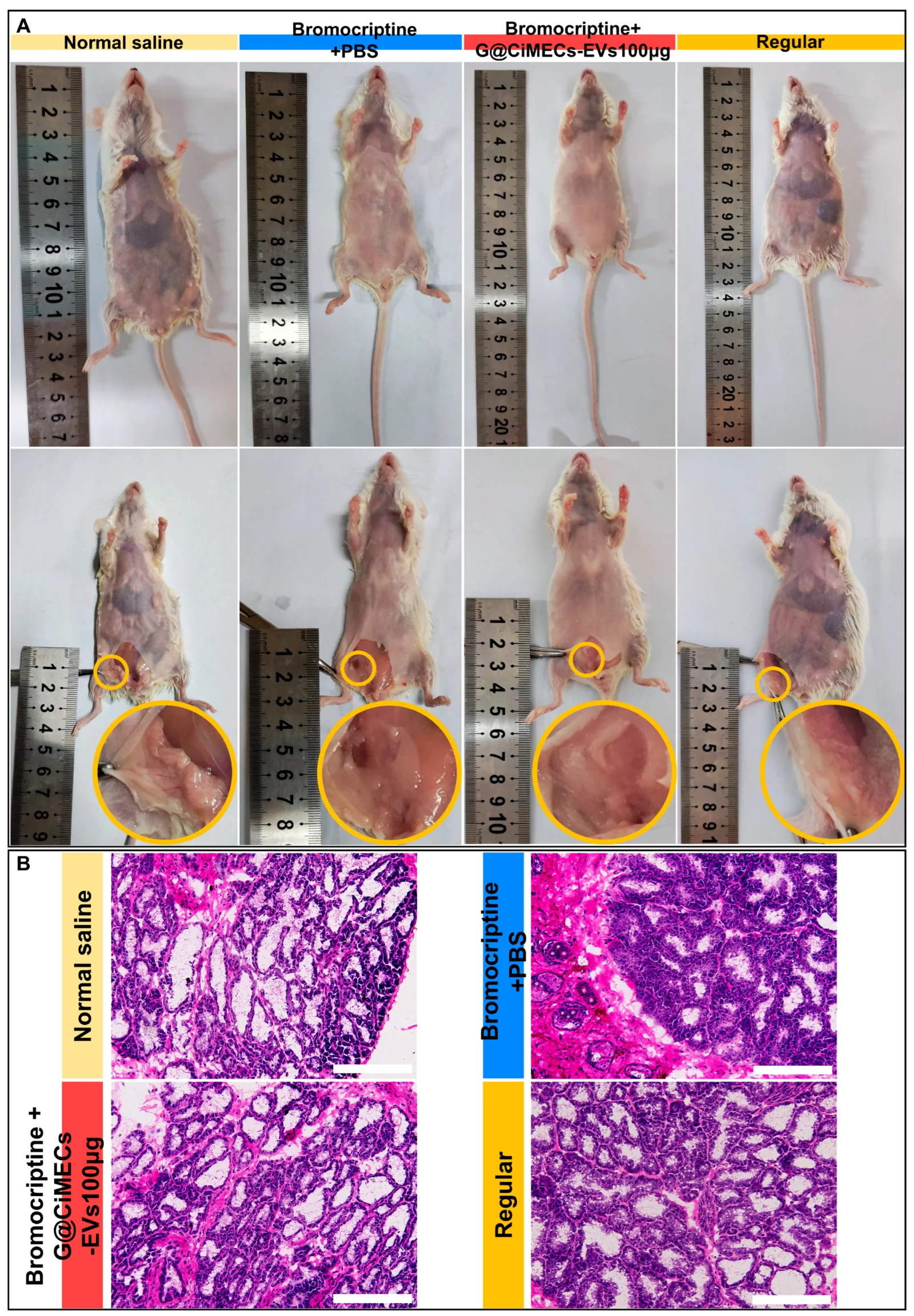
GelMA@CiMECs-EVs demonstrate a pronounced affinity for mammary tissue and exhibit biological safety. (**A**) Gross appearance of mammary glands in each group and subcutaneous morphological presentation of breast tissues. Yellow circles indicate the corresponding regions for magnified views. (**B**) Breast HE staining in each group. Compared with normal saline group, the bromocriptine + PBS group had retraction and stenosis of mammary gland ducts, indicating successful modeling of the lactation deficiency model. Among the bromocriptine + GelMA@PKH26-CiMECs-EVs-100 μg group and the regular group, the mammary gland duct structures in the two groups were smooth and full, indicating treatments were effective.

In terms of mammary gland index and macroscopic appearance, the 100 μg group showed consistent performance with the normal saline and regular groups. Moreover, compared with the model and GelMA groups, the mammary gland index was significantly elevated in the GelMA@CiMECs-EVs group (Figure 6E(a)), with plump physical status and full nipple morphology observed in mice (Figure 7A). In conclusion, 100 μg of GelMA@CiMECs-EVs can effectively ameliorate lactation function in lactation-deficient mice.

### 3.6. GelMA@CiMECs-EVs Demonstrate a Pronounced Affinity for Mammary Tissue and Exhibit Biological Safety

In order to further verify the safety of GelMA@CiMECs-EVs, this study also detected the index and HE staining of the liver, kidney, ovary, and uterus of all groups, and the results showed no significant differences among the six groups (Figure 6E(b–e); Figure S9), indicating that GelMA@CiMECs-EVs have biological safety in vivo.

**Figure 8:**
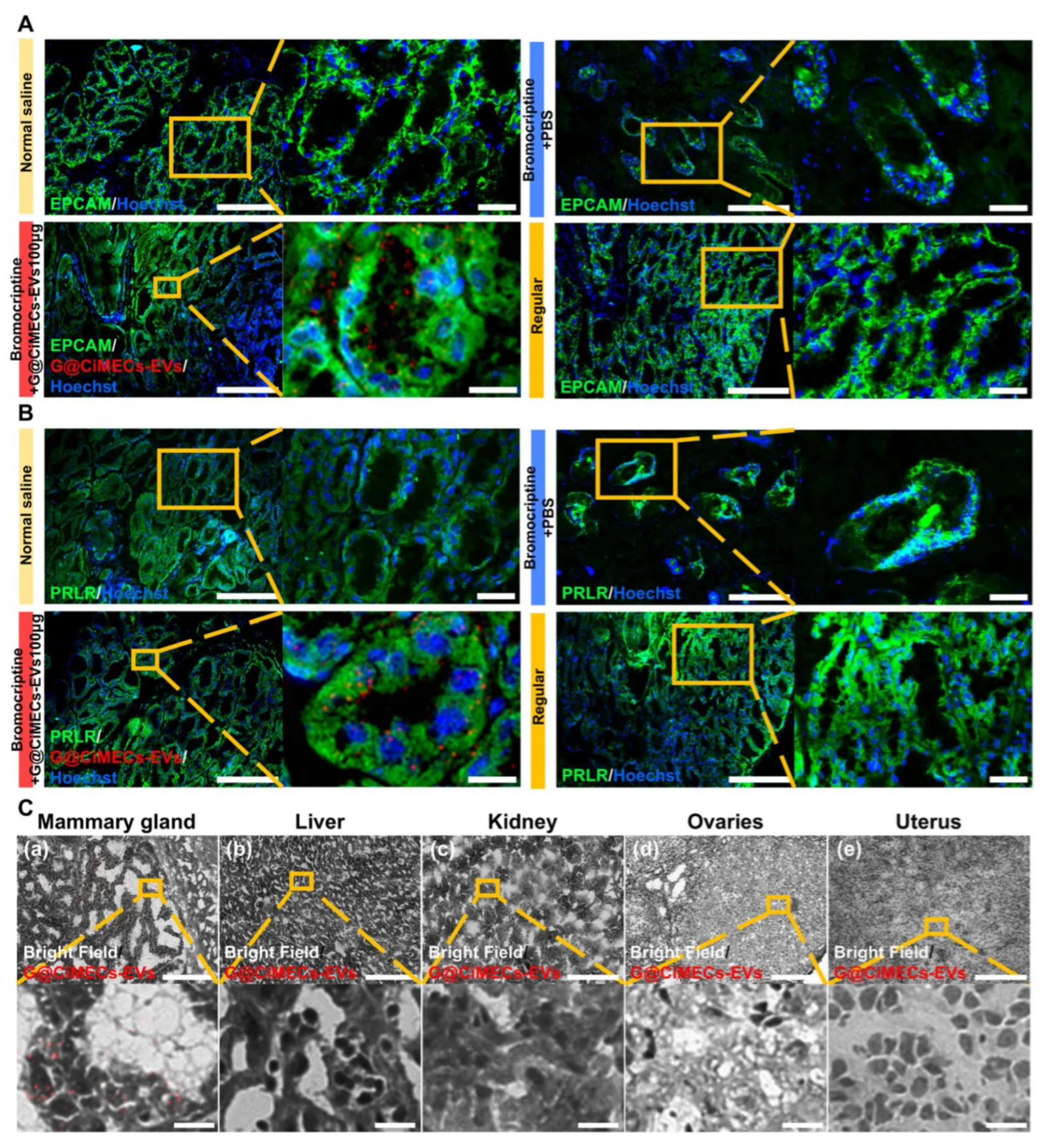
GelMA@CiMECs-EVs can restore lactation ability and have good homing ability in lactation-deficient mice. (**A**) EPCAM immunofluorescence staining of breast epithelial labeled protein in each group. The scale bars for the normal saline group, the bromocriptine + PBS group and the regular group are all 200 μm. The magnification scale bars are 40 μm. The scale bars for the bromocriptine + GelMA@CiMECs-EVs-100 μg group are 200 μm. The magnification scale bars are 10 μm. (**B**) PRLR immunofluorescence staining of breast epithelial labeled protein in each group. The scale bars for the normal saline group, the bromocriptine + PBS group and the regular group are all 200 μm. The magnification scale bars are 40 μm. The scale bars for the bromocriptine + GelMA@CiMECs-EVs-100 μg group are 200 μm. The magnification scale bars are 10 μm. (**C**) GelMA@CiMECs-EVs have good homing ability in lactation-deficient mice. The bromocriptine + GelMA@PKH26-CiMECs-EVs-100 μg group was examined for (**a**) breast, (**b**) liver, (**c**) kidney, (**d**) ovary, and (**e**) uterus retention after 14 days of treatment. Red fluorescence was seen only in the mammary gland, indicating that GelMA@PKH26-CiMECs-EVs did not spread significantly to other organs. Yellow boxes denote the selected regions for magnified views. (Scale bars, 200 μm. Magnification scale bars, 10 μm.)

Subsequently, to further clarify the therapeutic effects, the mammary glands of the above groups were subjected to HE staining and immunofluorescence detection. HE staining (Figure 7B; Figure S10B–D) showed that the mammary ducts in the GelMA-only group exhibited significant retraction and stenosis, similar to the model group. The free CiMECs-EVs group showed moderate ductal recovery but was notably inferior to the GelMA@CiMECs-EVs group, which exhibited smooth, full ductal structures comparable to the normal saline and regular groups. Immunofluorescence results (Figure 8A,B; Figure S10D) demonstrated that the expressions of EPCAM and PRLR were significantly lower in the GelMA-only and model groups, moderately expressed in the free EVs group, and highly expressed in the GelMA@EVs group. To further quantitatively validate these observations, we performed mean fluorescence intensity (MFI) analysis on the immunofluorescence images. As shown in Figure S11D, the MFI values of both EPCAM and PRLR were significantly higher in the GelMA@CiMECs-EVs group compared with the model group (*p* < 0.001), whereas the GelMA group showed no significant difference (ns). These quantitative data provide rigorous statistical support for our visual observations.

In order to further evaluate the safety of the treatment, organs of mice 14 days after treatment were examined, and it was found that GelMA@CiMECs-EVs resided in the mammary glands (red fluorescence) but not the liver, kidney, ovary, or uterus (Figure 8C). The above results indicate that GelMA@CiMECs-EVs have marked mammary tropism and do not significantly diffuse to other organs, further proving their safety and applicability.

### 3.7. GelMA@CiMECs-EVs Mitigate Mammary Oxidative Stress In Vivo

To validate the bioinformatics predictions suggesting that CiMECs-EVs possess abundant antioxidant cargo, we next performed quantitative biochemical assays to directly assess the oxidative stress status in the mammary glands of mice. As shown in Figure S10A, the model group exhibited significantly elevated levels of ROS and MDA, indicating intense oxidative damage (*p* < 0.001 vs. normal saline group). Concurrently, this was accompanied by a severe depletion of endogenous antioxidant defenses, evidenced by markedly decreased T-SOD, CAT, and GSH-Px activities, as well as a significant reduction in the GSH/GSSG ratio. Strikingly, the administration of GelMA@CiMECs-EVs prominently attenuated these pathological impairments. The levels of ROS and MDA were significantly decreased, while the activities of T-SOD, CAT, GSH-Px, and the GSH/GSSG ratio were all substantially elevated to levels close to those observed in the normal saline group (*p* < 0.001 compared with the model group). In contrast, the GelMA alone group produced no notable biochemical changes compared with the model group, confirming the biological inertness of the hydrogel. Moreover, when compared with the free CiMECs-EVs treatment (which only partially ameliorated the oxidative stress), the GelMA hydrogel-based delivery system demonstrated significantly superior therapeutic efficacy. These findings offer compelling biochemical evidence that the GelMA@CiMECs-EVs composite system effectively protects mammary tissues from oxidative injury by scavenging ROS, suppressing lipid peroxidation, and restoring the endogenous antioxidant enzyme system and redox homeostasis.

## 4. Discussion

### 4.1. Conceptual Innovation: Pioneering Discovery of CiMECs-EVs’ Inductive Capacity

Extracellular vesicles have the unique characteristics of low immunogenicity, biodegradability, high load, and facile cellular uptake, positioning them as a potential efficient and safe therapeutic tool [33,34,35,36]. In this study, the innovative isolation of CiMECs-EVs reveals a breakthrough: this unique EV subset enables the fate conversion of fibroblasts into mammary epithelial cells through the coordinated regulation of miRNAs and metabolites—a capability never before reported for EVs. No prior literature has demonstrated EVs’ ability to induce fibroblast transdifferentiation, marking this as a conceptual leap in understanding EV-mediated cell fate modulation. Thus, it provides a potential innovative strategy for the protection and improvement of breast structure and function.

Nevertheless, in future investigations, it would be highly valuable to further verify the functional specificity of CiMECs-EVs by directly comparing them with EVs derived from non-induced fibroblasts (GEFs) or native mammary epithelial cells (GMECs). Such a comparison would definitively validate the unique molecular cargo and lactogenic activity conferred by the chemical transdifferentiation process. Currently, the specific lactogenic effect of CiMECs-EVs is supported by their dose-dependent induction of fibroblasts in vitro (Figure 1B–D) and their superior in vivo therapeutic efficacy over free CiMECs-EVs or the GelMA matrix alone (Figure 6 and Figure 7).

The current study primarily focused on standard positive EV markers and physical characteristics for EV identification. We acknowledge that future work incorporating negative EV markers (e.g., Calnexin or GM130) and comparative analysis with donor-cell lysates would provide an even more definitive assessment of EV purity and cargo specificity.

### 4.2. Mechanistic Innovation: Multi-Omics Unveiling of Key Regulatory Molecules

We elucidated the molecular underpinnings of CiMECs-EVs’ function via integrated miRNA-seq, mRNA-seq, and metabolome analyses. The combined analysis showed that miR-126-5p was closely associated with key mammary epithelial cell regulators (LTF, GHR, EGFR) [37], while miR-30a-5p was correlated with PRLR and ITGA6 [38]. Moreover, this study is the first to link miR-126-5p to the TGFβR1/SMAD3 signaling axis—a previously identified driver of mammary epithelial cell fate in our laboratory [16,32]—establishing a clear mechanistic bridge between EV cargo and cellular reprogramming. Additionally, metabolome analysis revealed significantly enriched pathways (prolactin metabolism, galactose metabolism, tyrosine metabolism, lysine biosynthesis) closely tied to milk component synthesis [39,40,41], further validating that CiMECs-EVs orchestrate both transcriptional and metabolic rewiring for functional mammary epithelial cell generation. This redox imbalance framework is consistent with recent findings that oxidative stress critically impairs lactation performance in postpartum mice [42]. This multi-omics integration represents a mechanistic breakthrough in deciphering EV-driven lactation restoration.

It is important to clarify the regulatory direction of the key miRNAs identified in this study. Our dual-luciferase assays confirmed that miR-126-5p directly targets *TGFβR1* and *EGFR*, and miR-30a-5p targets *PRLR*. Since miRNAs typically suppress their direct targets, the observed upregulation of epithelial terminal genes (e.g., *PRLR, EGFR*, and *ITGA6*) during phenotypic induction might appear paradoxical. However, this can be explained by the network-level suppression mediated by miR-126-5p on the TGFβR1/SMAD3 inhibitory axis. By suppressing *TGFβR1/SMAD3*, miR-126-5p relieves the intrinsic repression of the mammary epithelial fate, thereby overriding the direct suppressive effects on terminal epithelial markers and leading to a net upregulation of these genes necessary for the functional transition. We acknowledge that direct quantification of miRNA levels in donor cells and recipient fibroblasts, as well as engineering donor cells to alter EV cargo, would provide even more definitive transfer evidence. We plan to conduct these experiments in our future mechanistic studies.

### 4.3. Technological Innovation: GelMA-Encapsulation System for Sustained Efficacy

A major technical challenge in EV-based therapies is their structural fragility and insufficient controlled-release profile, which significantly restricts long-term therapeutic outcomes [43,44]. To overcome this limitation, we developed a GelMA hydrogel-based delivery system to encapsulate CiMECs-EVs. This innovative strategy not only provides physical protection for EVs but also facilitates their durable and controlled release via the three-dimensional polymeric network. In contrast to traditional free EVs (e.g., MSC-EVs, which suffer from poor tissue retention [45,46]), our GelMA@CiMECs-EVs platform effectively circumvents rapid in vivo clearance while preserving EV bioactivity, thereby substantially extending their therapeutic window within mammary tissue. Consequently, this technological advancement not only boosts the therapeutic performance of CiMECs-EVs but also establishes a versatile and robust scaffold for EV-based regenerative applications.

Furthermore, this three-dimensional hydrogel microenvironment is known to influence cellular plasticity and facilitate the mesenchymal-to-epithelial transition, as demonstrated in recent studies utilizing 3D hydrogels for somatic cell reprogramming [47].

The robust recovery of antioxidant enzyme activities and GSH/GSSG balance provides strong evidence that the sustained delivery of CiMECs-EVs through GelMA effectively alleviates mammary oxidative stress.

Notably, the unexpected rheological and degradation shifts observed at the highest EV concentration (500 µg) compared with the 100 µg group in this study can be comprehensively explained by physicochemical interferences within the hydrogel network. First, the high density of EVs physically occupies the interstitial spaces of the GelMA polymer chains, acting as steric hindrance that obstructs the free radical polymerization process, thereby leading to a less dense crosslinking network. Second, the highly turbid dispersion of 500 µg EVs scatters the incident 365 nm UV light used for photocrosslinking, which reduces the excitation efficiency of the LAP photoinitiator and further decreases the effective crosslinking density within the hydrogel matrix. Finally, the abundant protein and lipid cargoes of the concentrated EVs compete for water binding sites within the hydrophilic GelMA network, altering the hydration state of the hydrogel and consequently affecting its viscosity and degradation profile. These mechanistic explanations align well with the macroscopic behaviors observed in Figure 5C–E, where the 500 µg group exhibited a sparser viscoelastic network and slower hydration/degradation dynamics.

It is important to note that we have not yet performed direct experimental comparisons to evaluate the potential effects of UV crosslinking on EV structural integrity and cargo stability. Nevertheless, the sustained release profile observed in Figure 5F and the significant in vivo therapeutic outcomes in Figure 6 strongly suggest that the loaded CiMECs-EVs retained their biological activity throughout the photocrosslinking and subsequent in vivo delivery processes. Systematic investigations into the direct impact of UV exposure on EV integrity will be a valuable focus in our future studies.

We acknowledge that an extended in vitro release study beyond 24 h and a direct in vivo comparison using a single administration of GelMA@CiMECs-EVs versus free CiMECs-EVs would provide more definitive evidence for long-term sustained-release benefits. Although our current study employed daily repeated dosing to ensure continuous local EV concentration against persistent oxidative stress, our existing comparative data consistently demonstrated that the GelMA-encapsulated group significantly outperformed the free EV group over the 10-day period, confirming that GelMA effectively mitigated rapid EV clearance in vivo. We plan to conduct more extensive release kinetics and single-dose pharmacokinetic studies in our future investigations.

### 4.4. Broader Application Prospects and Interdisciplinary Impact

Beyond postpartum hypogalactia, the GelMA@CiMECs-EVs platform holds significant potential in post-breast cancer reconstruction—a clinical scenario where breast tissue structure and lactation function are often compromised. EVs have been shown to promote wound healing and tissue regeneration in breast surgery models [48,49], and our system’s ability to induce mammary epithelial cell fate and provide 3D structural support via GelMA could facilitate functional breast tissue regeneration post-mastectomy or lumpectomy.

Additionally, this platform’s design is generalizable to other tissue engineering fields. For instance, in skin wound repair, GelMA hydrogels loaded with EVs have been reported to accelerate healing by modulating inflammation and promoting angiogenesis; similarly, our system could be adapted for cutaneous regeneration by replacing CiMECs-EVs with skin-specific EVs. In cartilage regeneration, adjusting GelMA’s mechanical properties to match the cartilage microenvironment while encapsulating chondrocyte-derived EVs could offer a novel strategy for articular tissue repair [49]. These prospects underscore the interdisciplinary value of our research, positioning GelMA@EVs as a versatile tool for regenerative medicine across multiple tissues.

In summary, our study innovatively presents CiMECs-EVs as a tool to modulate mammary epithelial cell fate and ameliorate lactation, with the GelMA encapsulation system enabling sustained efficacy. This work not only addresses a critical clinical need in postpartum care but also lays the groundwork for broader applications in tissue engineering and regenerative medicine.

## Figures and Tables

**Table 1:** Key resources table.

Reagent or Resource	Source	Identifier
Antibodies		
Rabbit monoclonal anti-CD63	Abcam	Cat#ab134045; RRID: AB_2800495
Mouse monoclonal anti-CD81	NOVUS	Cat#NB100-65805; RRID: AB_962702
Rabbit polyclonal anti-TSG101	Sigma-Aldrich	Cat#AV38773; RRID: AB_1858389
Rabbit monoclonal anti-CDH1	Abcam	Cat#ab40772; RRID: AB_731493
Rabbit monoclonal anti-EPCAM	Abcam	Cat#ab223582; RRID: AB_2762366
Mouse monoclonal anti-KRT19	Abcam	Cat#ab7754; RRID: AB_306048
Mouse monoclonal anti-ITGA6	Abcam	Cat#ab20142; RRID: AB_445361
Rabbit monoclonal anti-KRT8	Abcam	Cat#ab53280; RRID: AB_869901
Rabbit monoclonal anti-Vimentin	Abcam	Cat#ab92547; RRID: AB_10562134
Alexa Fluor 488 donkey anti-mouse	Abcam	Cat#ab150109; RRID: AB_2571721
Alexa Fluor 555 donkey anti-rabbit	Abcam	Cat#ab150074; RRID: AB_2636997
Chemicals, Drugs and Reagents		
RepSox	Selleck	S7223; CAS:446859-33-2
TTNPB	Selleck	S4627; CAS:71441-28-6
Forskolin	Selleck	S2449; CAS:66575-29-9
Tranylcypromine	Selleck	S4246; CAS:1986-47-6
VPA	Selleck	S3944; CAS:99-66-1
Bromocriptine	Gedeon Richter	CAS:62675-96-9
GelMA	Engineering For Life	EFL-GM-30
PKH26	Sigma	PKH26GL
Critical Commercial Assays		
RNA isolator Total RNA Extraction Reagent	Vazyme	Cat#R401-01
HiScript III RT SuperMix for qPCR kit	Vazyme	Cat#R323-01
ChamQ SYBR qPCR Master Mix kit	Vazyme	Cat#Q711-02
Experimental Models: Goat ear margin fibroblasts were obtained by primary isolation in our laboratory.		
Oligonucleotides		
Primer: *Col6a2* Forward: GACCATCCCCCTGATCCAAC	This paper	N/A
Primer: *Col6a2* Reverse: TCGTTTCCTGTGGCGAACTT	This paper	N/A
Primer: *Fbn1* Forward: CATATCCATCGCGGGAACCA	This paper	N/A
Primer: *Fbn1* Reverse: CCTTTGTTGCACTCACACCG	This paper	N/A
Primer: *Vimentin* Forward: TCTGAAGCTGCTAACCGCAA	This paper	N/A
Primer: *Vimentin* Reverse: TCTGAAGCTGCTAACCGCAA	This paper	N/A
Primer: *Fibin* Forward: GTCAGGGCTATTTCGACGGT	This paper	N/A
Primer: *Fibin* Reverse: CAGGGTGAGGCTTAGCAGAG	This paper	N/A
Primer: *Krt19* Forward: CTCCGGGCATCGACCTAGCCAA	This paper	N/A
Primer: *Krt19* Reverse: CTCCTTGTTCAGCTCCTCGGTCT	This paper	N/A
Primer: *Itga6* Forward: AGCCAATCATAGTGGAGCTGT	This paper	N/A
Primer: *Itga6* Reverse: TCTTGCCACCCGTCTTTGTT	This paper	N/A
Primer: *Cdh1* Forward: ACAATGACCAAGAACGTGAGT	This paper	N/A
Primer: *Cdh1* Reverse: TGCACATCAAGTGTAGTCACC	This paper	N/A
Primer: *Epcam* Forward: CCACCTGAATTTTCCATGCAG	This paper	N/A
Primer: *Epcam* Reverse: GGACACAACCAGCACAATGAT	This paper	N/A
Primer: *Gapdh* Forward: CGTTGCCATCAATGACCCCTT	This paper	N/A
Primer: *Gapdh* Reverse: CGTACTCAGCACCAGCATCACC	This paper	N/A
Software, Algorithms and Website		
GraphPad Prism 9.5.0	GraphPad Software	https://www.graphpad.com/, accessed on 12 March 2025
ImageJ 1.54p	NIH	https://imagej.nih.gov/ij/download.html, accessed on 27 April 2025
R version 4.0.5	The R Foundation for Statistical Computing	https://www.r-project.org/, accessed on 8 May 2025
miRTarBase 2025 (v10.0)	miRNA prediction	https://mirtarbase.cuhk.edu.cn/, accessed on 19 June 2025
TargetScan Human 7.1	miRNA prediction	https://www.targetscan.org/vert_71/, accessed on 3 July 2025
miRDB v6.0	miRNA prediction	https://mirdb.org/mirdb/index.html, accessed on 22 July 2025
STRING-db v12.0	Protein Interaction Network Analysis	https://string-db.org/, accessed on 14 August 2025

**Table 2:** Experimental grouping.

Group Name	Group Notes
Normal saline	Gavage of saline to form a control between the groups with bromocriptine.
Bromocriptine + PBS	Breast deficiency model (gavage of bromocriptine) + PBS treatment at the breast site
Bromocriptine + GelMA	Lactation-abscess model (gavage of bromocriptine) + mammary site injection of blank GelMA hydrogel (GelMA control group)
Bromocriptine + CiMECs-EVs (100 μg)	Lactation-abscess model (gavage of bromocriptine) + mammary site injection of free CiMECs-EVs (100 µg) without GelMA encapsulation (Free-EVs group)
Bromocriptine + GelMA@CiMECs-EVs (100 μg)	Lactation-abscess model (gavage bromocriptine) + mammary site injection of 100 μg of GelMA@CiMECs-EVs treatment
Regular	Normal pregnancy and delivery group

## Data Availability

The original data presented in the study are openly available in the NCBI Gene Expression Omnibus (GEO) database at [https://www.ncbi.nlm.nih.gov/geo/query/acc.cgi?acc=GSE342949 and https://www.ncbi.nlm.nih.gov/geo/query/acc.cgi?acc=GSE342950] (accessed on 1 June 2026, accession numbers GSE342949 and GSE342950). Further information and requests for resources and reagents should be directed to and will be fulfilled by the lead contact, Ben Huang.

## Supplementary Materials

The following supporting information can be downloaded at: https://www.mdpi.com/article/10.3390/antiox15091115/s1, Figure S1: RepSox-induced fibroblast transdifferentiation into chemically induced mammary epithelial cells (CiMECs); Figure S2: mRNA-Seq demonstrates that RepSox can induce fibroblasts to become mammary epithelial cells; Figure S3: mRNA-Seq demonstrates that RepSox can induce fibroblasts to become mammary epithelial cells; Figure S4: mRNA-Seq demonstrates that RepSox can induce fibroblasts to become mammary epithelial cells; Figure S5: tSNE, PCA and differential miRNA volcano maps of miRNA-Seq for CiMECs-EVs; Figure S6: GO Enrichment Analysis of Key miRNAs; Figure S7: KEGG Enrichment Analysis of Key miRNAs; Figure S8: Functional Validation of Key miRNAs; Figure S9: HE staining images of each group and each organ; Figure S10: Biochemical and histological characterization of the GelMA and free CiMECs-EVs control groups; Figure S11: Dual-luciferase reporter assay validation of miRNA-target interactions and quantitative immunofluorescence analysis; Table S1: miRNA sequences; Table S2: miRNA mimics and inhibitor sequences; Table S3: miRNA qPCR primers.
