# Supplementary material for: Gelatin Methacryloyl Hydrogel Encapsulating CiMECs-Derived Extracellular Vesicles Ameliorates Lactation Function via Alleviating Mammary Oxidative Stress
Source: Antioxidants (Basel). 2026 Sep 4;15(9):1115. doi: 10.3390/antiox15091115 (PMC13603849; doi:10.3390/antiox15091115)
Supplement: Supplementary file 1 [file antioxidants-15-01115-s001.zip › antioxidants-4428200-supplementary.pdf]

antioxidants

MDPI

Supplementary Materials

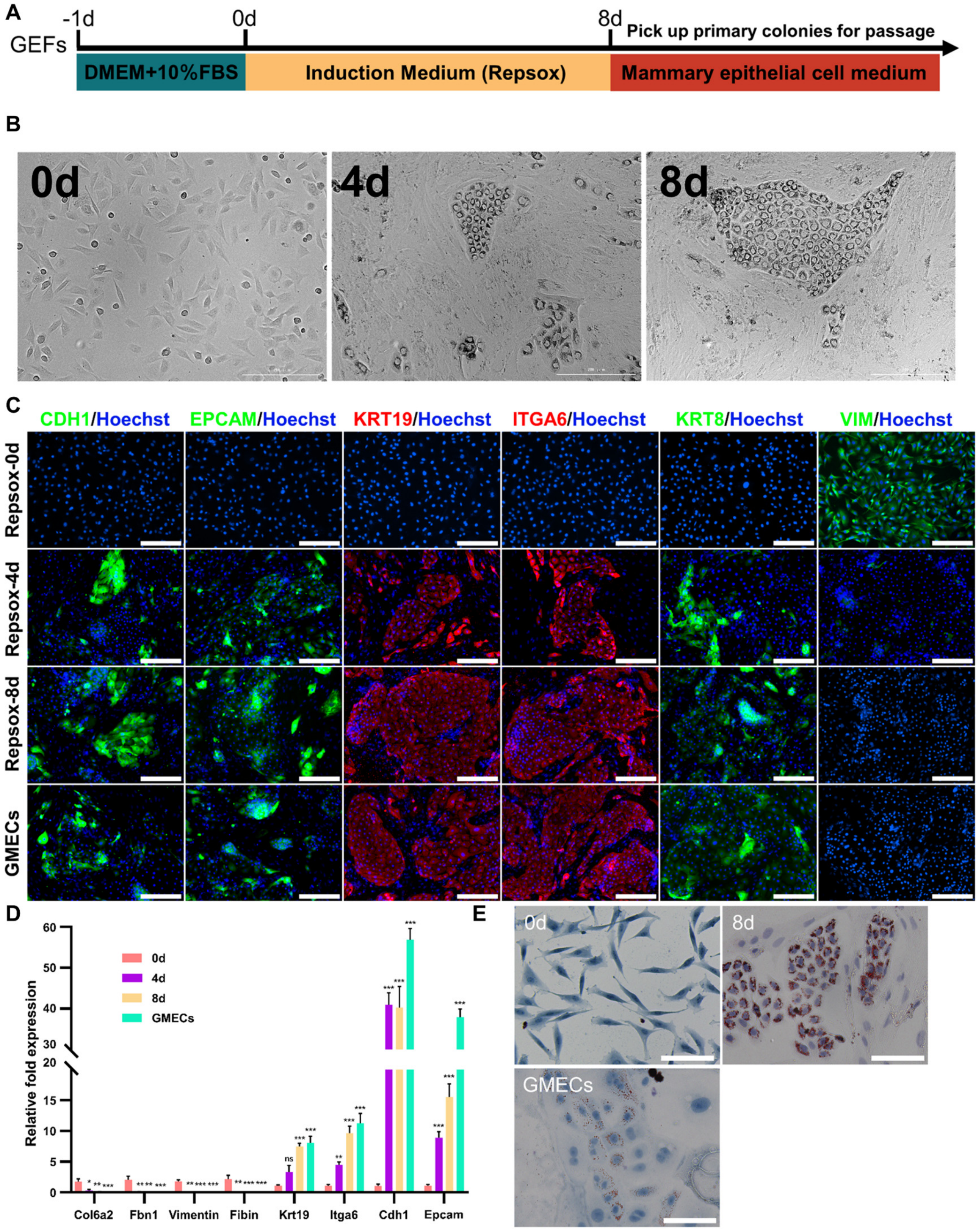

Figure S1. RepSox-induced fibroblast transdifferentiation into chemically induced mammary epithelial cells (CiMECs). A: The diagram of generation of CiMECs from fibroblast by RepSox induction. B: RepSox-induced Morphological changes of fibroblasts at 0d, 4d, 8d (Scale bars, 200 μm) C: RepSox-0d, -4d, -8d and immunofluorescence staining of mammary epithelial cell marker proteins (CDH1, EPCAM, KRT19, ITGA6, KRT8) and fibroblast marker proteins (VIM) of mammary epithelial cells (GMECs). Repsox-0d does not express mammary epithelial cell marker protein, but highly expresses fibroblast cell marker protein, RepSox-8d and goat mammary epithelial cells (GMECs) highly express mammary epithelial cell marker protein, but not fibroblast marker

protein (Scale bars, 200 μm). D: RepSox-0d, -4d, -8d and GMECs marker genes (*KRT19*, *ITGA6*, *CDH1*, *EPCAM*) and fibroblast marker genes (*COL6A2*, *FBN1*, *VIMENTIN*, *FIBIN*) quantitative PCR detection (mean ± SEM, n=3 biological replicates, *$p < 0.05$, **$p < 0.01$, ***$p < 0.001$, one-way ANOVA). E: The oil red O staining for RepSox-0d, -8d cells and GMECs (Scale bars, 100 μm).

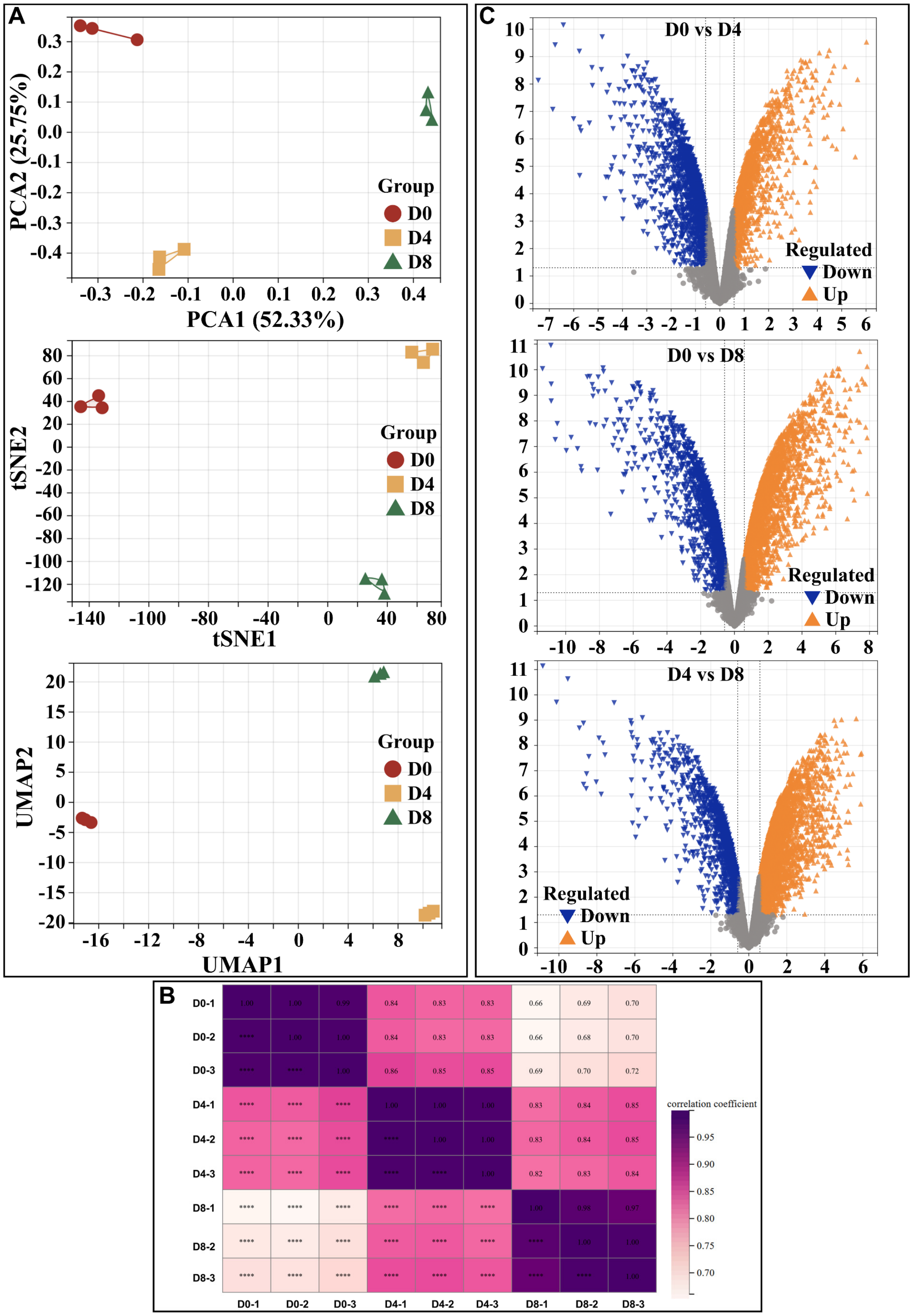

Figure S2. mRNA-Seq demonstrates that RepSox can induce fibroblasts to become mammary epithelial cells. A: UMAP, tSNE, PCA analysis verified the consistency of RepSox-0d, -4d, -8d samples. B: RepSox-0d, -4d, -8d sample correlation analysis. C: RepSox-0d, -4d, -8d differential gene analysis.

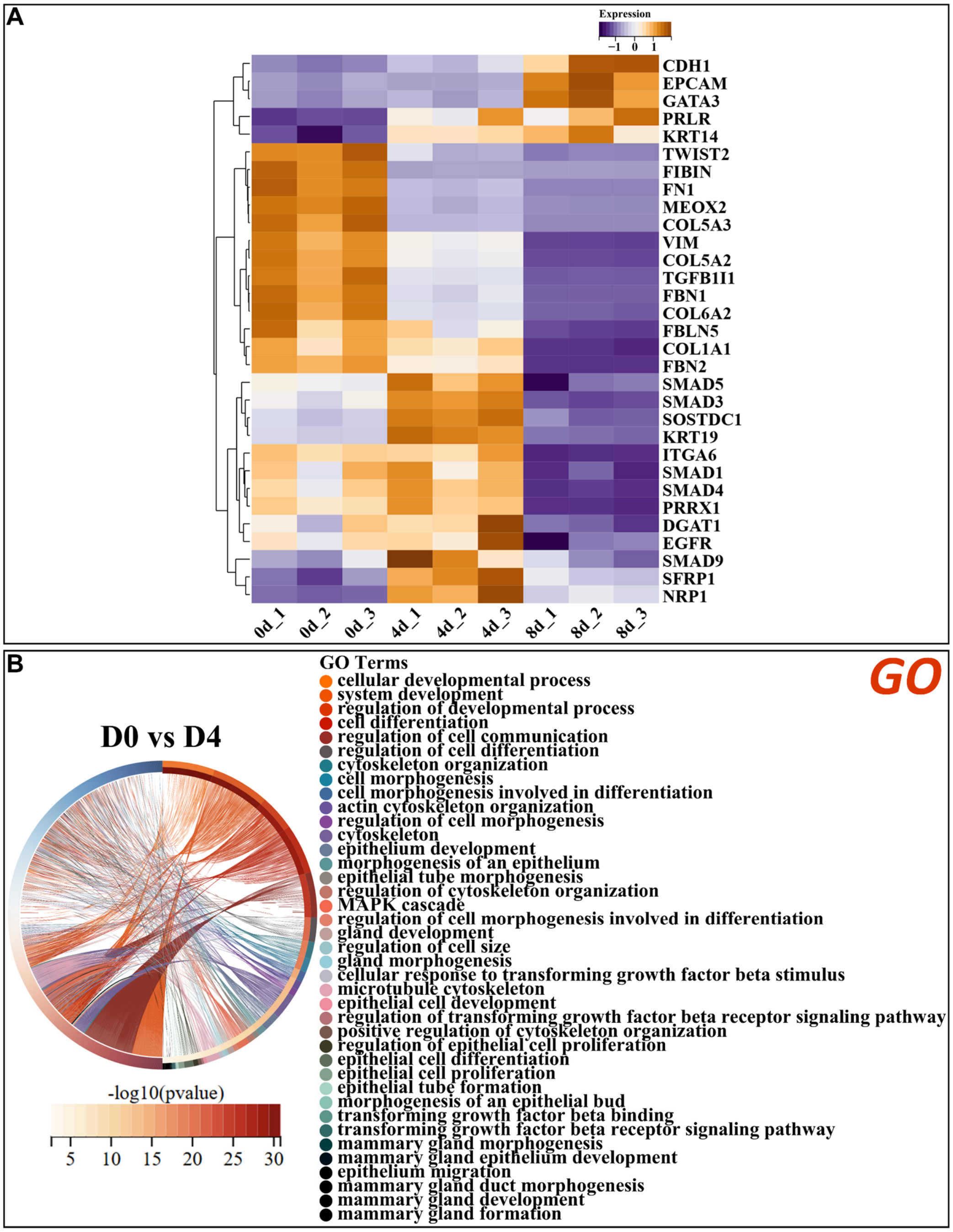

Figure S3. mRNA-Seq demonstrates that RepSox can induce fibroblasts to become mammary epithelial cells. A: RepSox-0d, -4d, -8d cluster heat maps showed that with the increase of induction time, the fibroblast fate gradually entered the mammary epithelial cell fate. B: GO enrichment analysis of RepSox-0d vs 4d differential genes. With the increase of induction time, GO entries and related pathways enriched into mammary epithelial cells gradually increased.

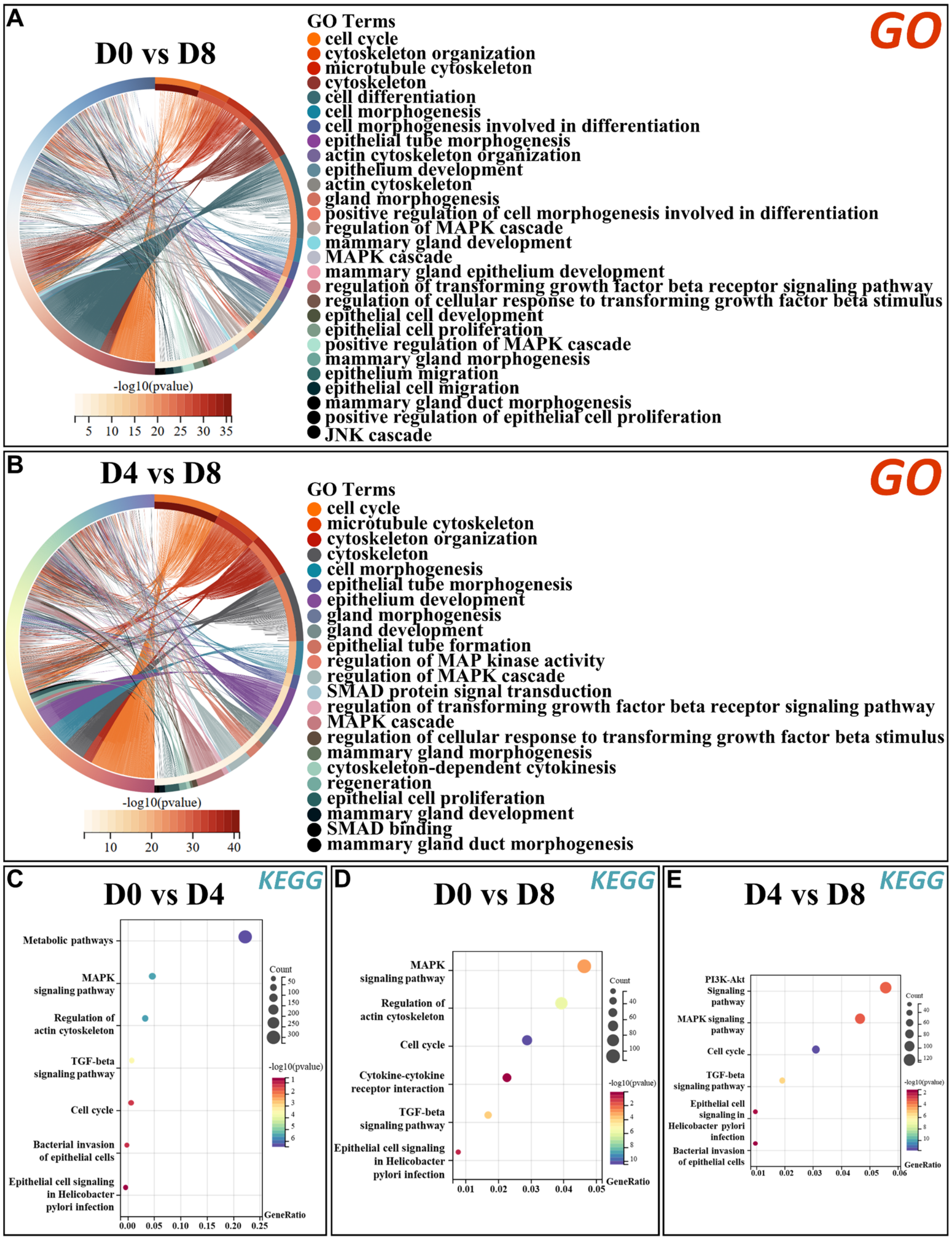

Figure S4. mRNA-Seq demonstrates that RepSox can induce fibroblasts to become mammary epithelial cells. A-B: GO enrichment analysis of RepSox-0d vs 8d, -4d vs 8d differential genes. With the increase of induction time, GO entries and related pathways enriched into mammary epithelial cells gradually increased. C-E: KEGG enrichment analysis of RepSox-0d vs 4d, -0d vs 8d, -4d vs 8d differential genes. With the increase of induction time, KEGG entries and related pathways enriched into mammary epithelial cells gradually increased.

https://doi.org/10.3390/antiox15091115

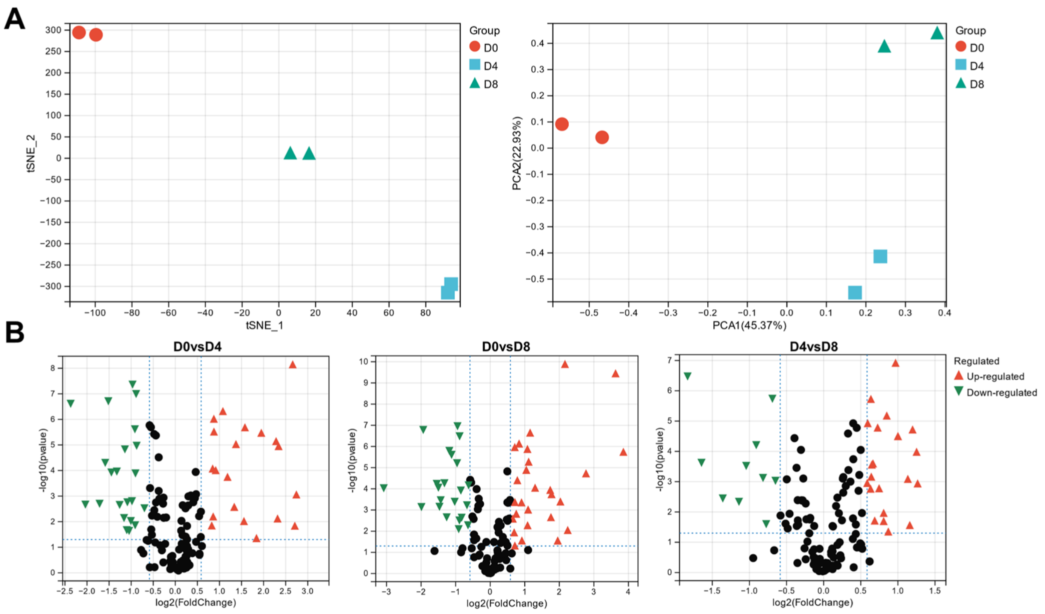

Figure S5. tSNE, PCA and differential miRNA volcano maps of miRNA-Seq for CiMECs-EVs. A: tSNE and PCA verified the sample consistency of CiMECs-EVs-0d, -4d and -8d in miRNA-Seq. B: The volcano map shows the differential miRNA of CiMECs-EVs-0d, -4d, -8d.

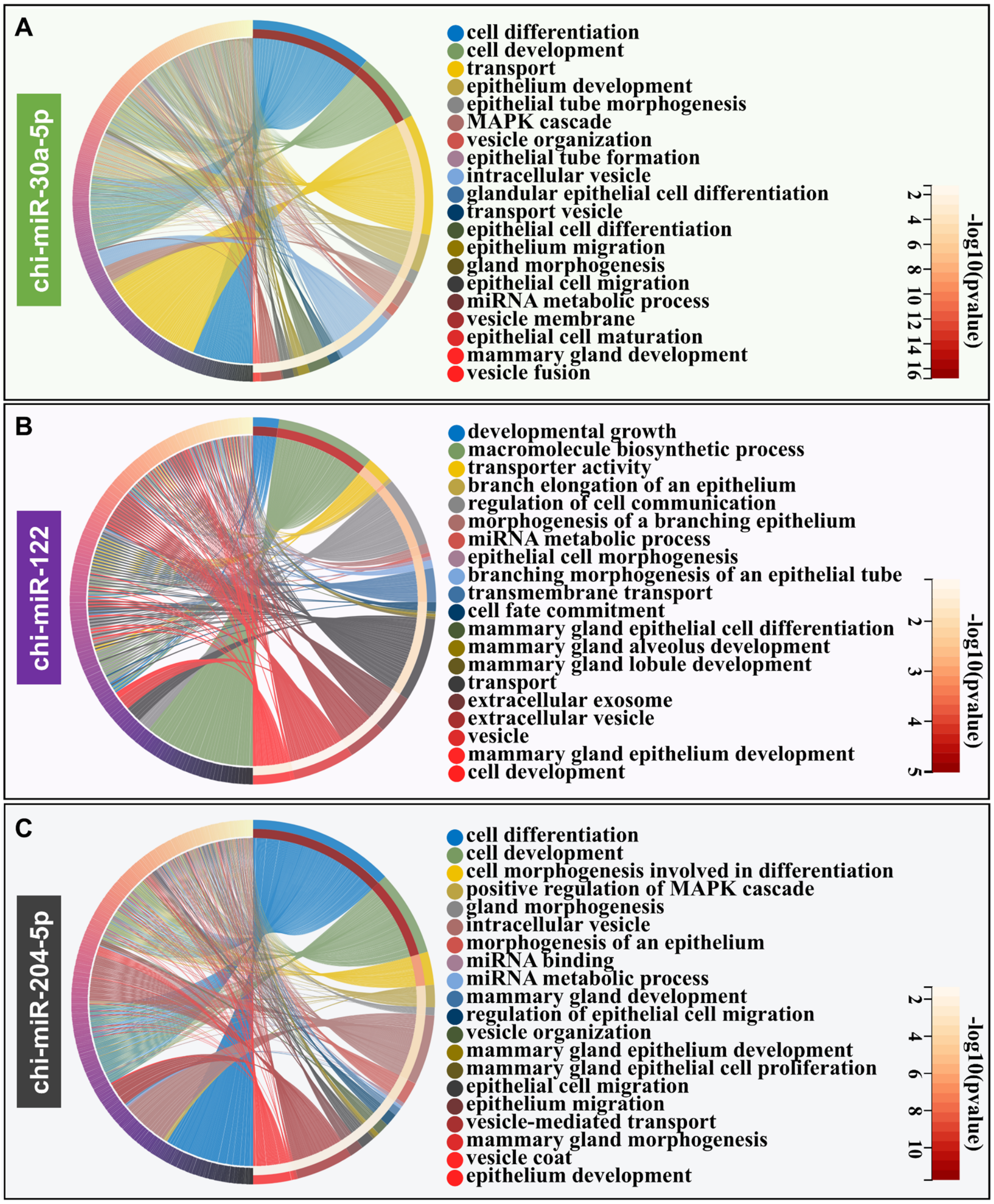

Figure S6. GO Enrichment Analysis of Key miRNAs. A-C: GO Enrichment Chord Diagram of chi-miR-30a-5p, chi-miR-122, and chi-miR-204-5p.

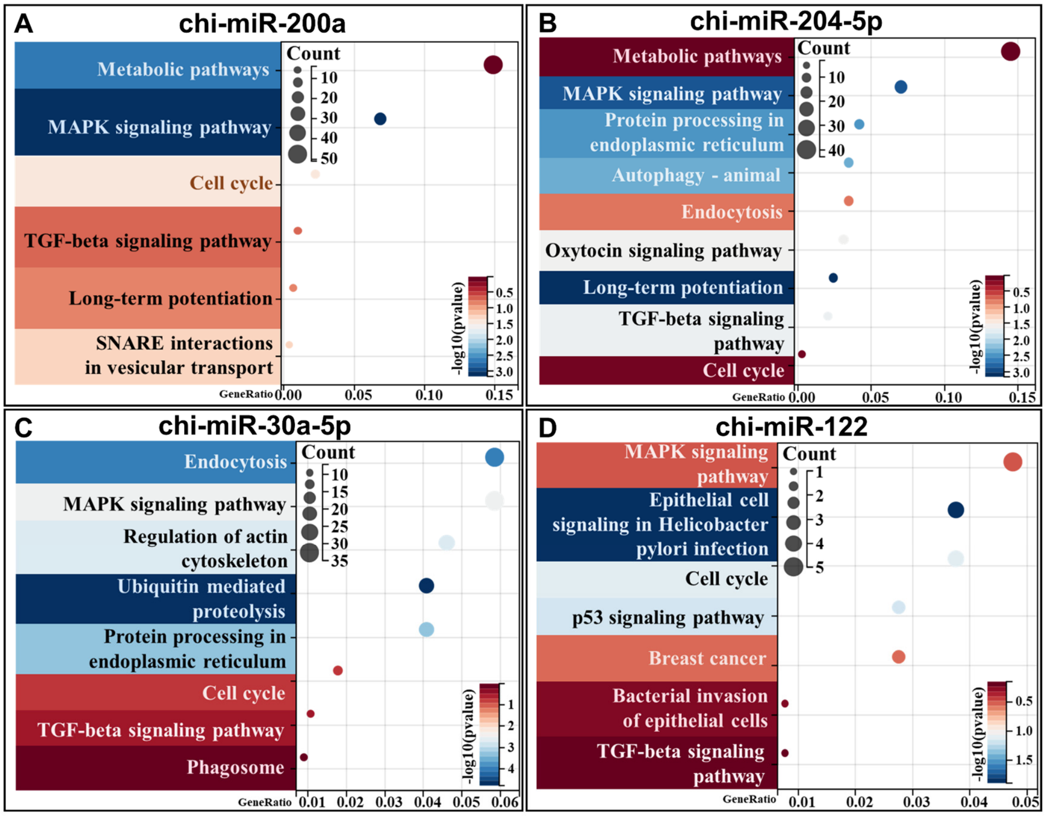

Figure S7. KEGG Enrichment Analysis of Key miRNAs. A-D: KEGG Enrichment Bubble Plot of chi-miR-200a, chi-miR-204-5p, chi-miR-30a-5p, and chi-miR-122.

https://doi.org/10.3390/antiox15091115

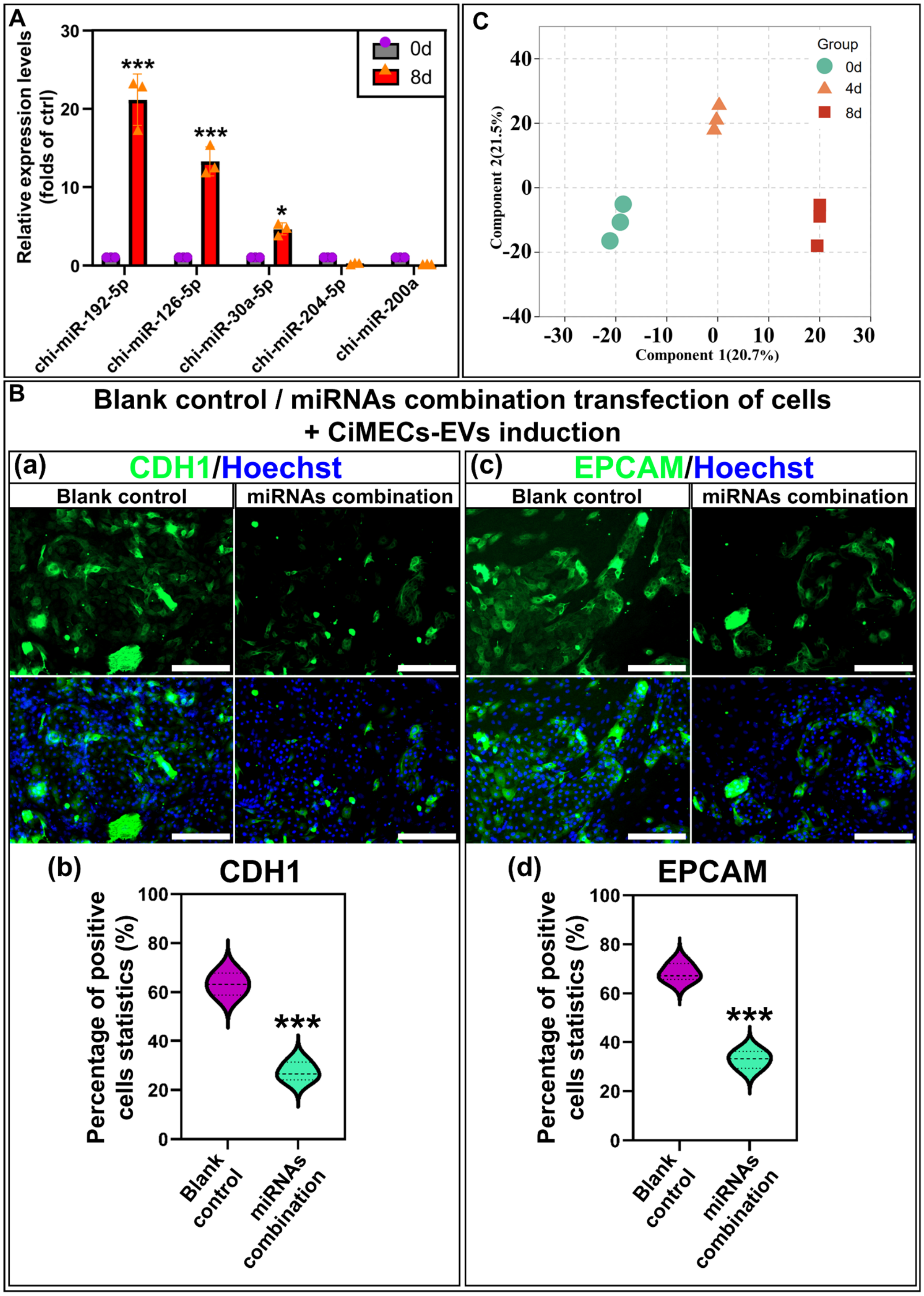

Figure S8. Functional Validation of Key miRNAs. A: qRT-PCR Detection of Key miRNAs.B: (a) Immunofluorescence detection of CDH1 antibody in mammary epithelial cells induced by Ci-MECs-EVs before and after transfection with the key miRNA combination; (b) Statistical graph of the CDH1 antibody-positive rate; (c) Immunofluorescence detection of EPCAM antibody in mammary epithelial cells induced by CiMECs-EVs before and after transfection with the key miRNA combination; (d) Statistical graph of the EPCAM antibody-positive rate (Scale bars, 200μm). C: CiMECs-EVs-0d, -4d, -8d metabolome OPLS-DA score map.

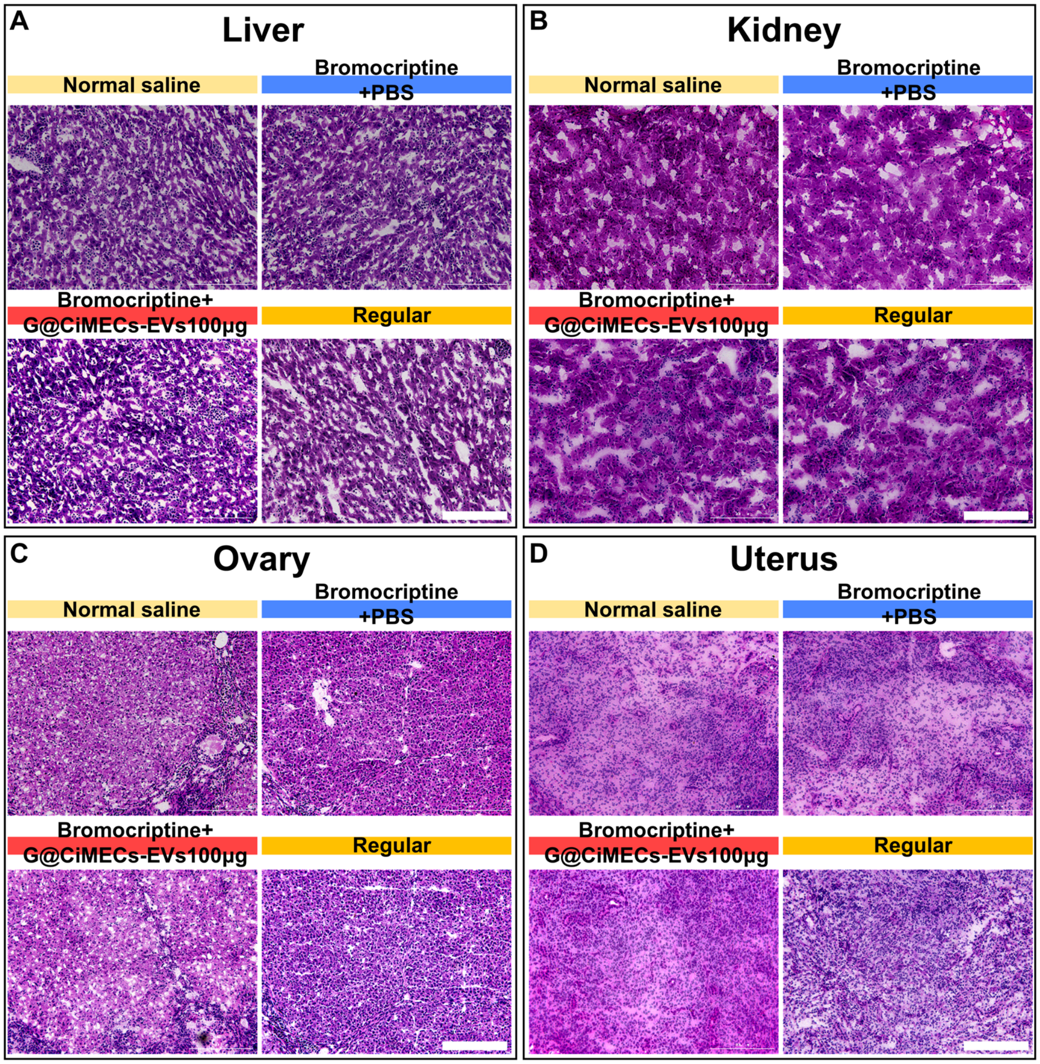

Figure S9. HE staining images of each group and each organ. A-D: The HE staining images of the liver, kidney, ovary and uterus of each group (Scale bars, 200 μm).

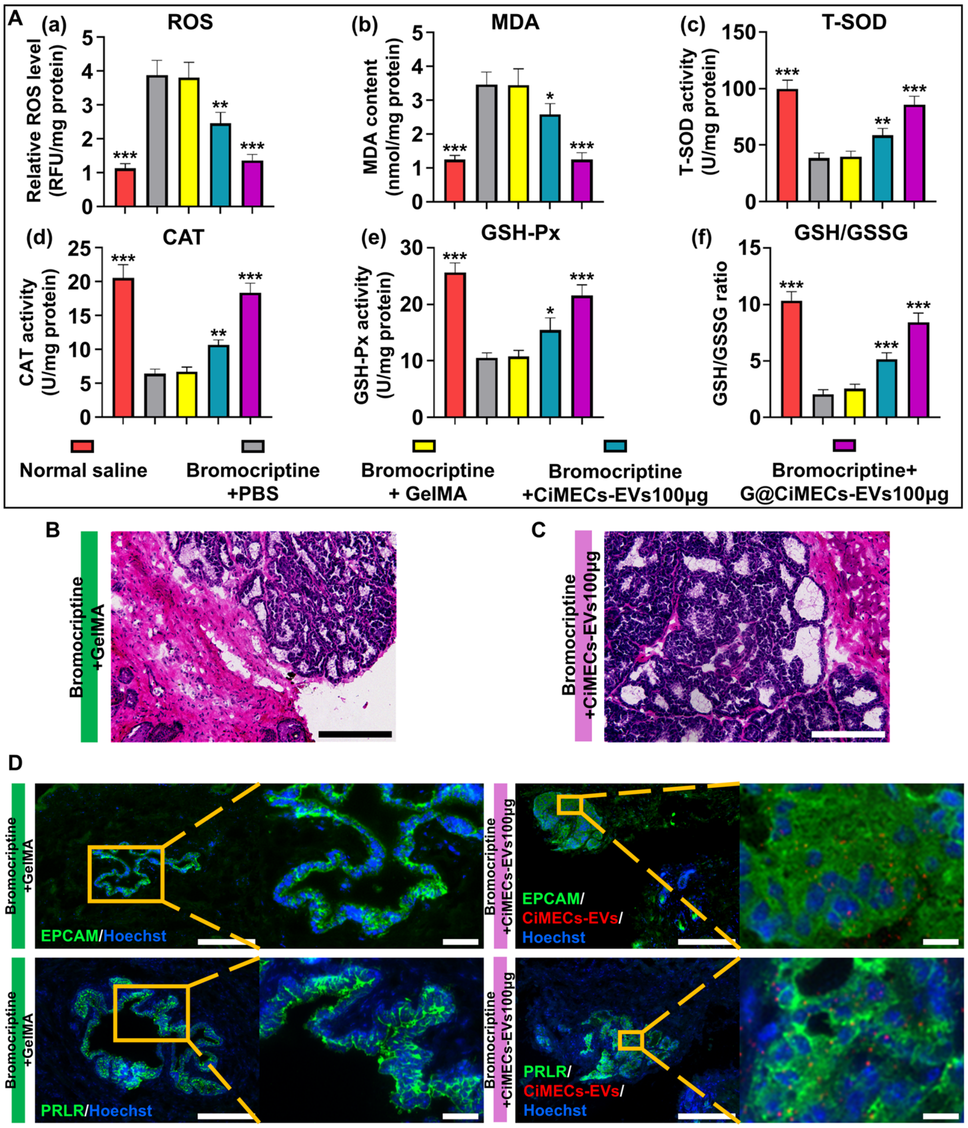

Figure S10. Biochemical and histological characterization of the GelMA and free CiMECs-EVs control groups. A: Quantitative analyses of (a) ROS levels, (b) MDA content, (c) T-SOD activity, (d) CAT activity, (e) GSH-Px activity, and (f) GSH/GSSG ratio in mammary gland tissues from the five experimental groups. Data are presented as mean ± SEM (n=6 individual mice per group). Statistical significance was evaluated by one-way ANOVA followed by Tukey's post hoc test. *p<0.05, **p<0.01, and ***p<0.001, compared with the Model group. B-C: Representative HE staining images of mammary gland tissues from the (B) Bromocriptine+GelMA group and (C) Bromocriptine+CiMECs-EVs group. Scale bars: 200 μm. D: Representative immunofluorescence images showing the expression of EPCAM and PRLR (green fluorescence) in the two control groups. Red fluorescence signals correspond to PKH26-labeled CiMECs-EVs. The scale bars for the low-magnification images are 200 μm for all groups. The magnification scale bars for the yellow-boxed regions are 40 μm for the Bromocriptine+GelMA group (left panels) and 10 μm for the Bromocriptine+CiMECs-EVs group (right panels). Note that the GelMA group exhibited ductal constriction and low protein expression similar to the Model group, while the free EVs group showed partial but insufficient recovery compared to the GelMA@CiMECs-EVs group shown in the main figures.

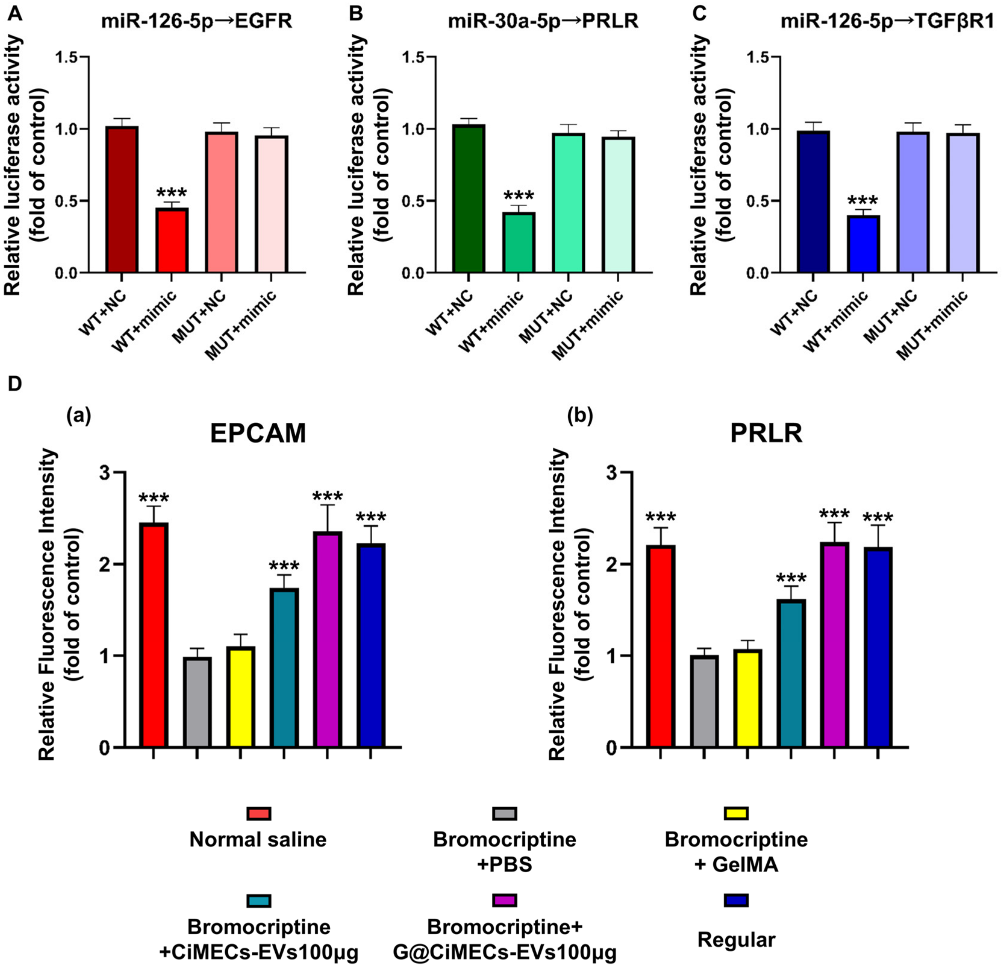

Figure S11. Dual-luciferase reporter assay validation of miRNA-target interactions and quantitative immunofluorescence analysis. (A-C) HEK293T cells were co-transfected with pmirGLO vectors containing either the wild-type (WT) or mutant (MUT) 3'UTRs of EGFR (A), PRLR (B), or TGFβR1 (C), along with specific miRNA mimics (chi-miR-126-5p or chi-miR-30a-5p) or negative control (NC). Relative luciferase activity was measured 48 hours post-transfection. (D) Quantitative analysis of the mean fluorescence intensity (MFI) of EPCAM and PRLR in mammary gland tissues from the six experimental groups. MFI values were quantified using ImageJ software from 6 independent visual fields per group. Data are presented as mean ± SEM. Statistical significance was evaluated by one-way ANOVA followed by Tukey's post hoc test. ***$p < 0.001$ compared with the WT+NC group or the Model group; "ns" indicates no significant difference.

Table S1. miRNA sequences.

chi-miR-204-5p	UUCCCUUUGUCAUCCUAUGCCU
chi-miR-200a	UAACACUGUCUGGUAACGAUG
chi-miR-192-5p	CUGACCUAUGAAUUGACAGCC
chi-miR-126-5p	CAUUAUUACUUUUGGUACGCGC
chi-miR-30a-5p	UGUAAACAUCCUCGACUGGAAGCU

Table S2. miRNA mimics and inhibitor sequences.

miRNA	mimic (5'→3')	Inhibitor (5'→3')

chi-miR-204-5p	F: UUCCCUUUGUCAUCCUAUGCCU<br>R: AGGCUAUGGAUGACAAAGGGAA	AGGCUAUGGAUGACAAAGGGAA
chi-miR-200a	F: UAACACUGUCUGGUAACGAUG<br>R: CAUCGUUACCAGACAGUGUUAU	CAUCGUUACCAGACAGUGUUAU
chi-miR-192-5p	F: CUGACCUAUGAAUUGACAGCC<br>R: GGCUGUCAAUUCAUAGGUCAGU	GGCUGUCAAUUCAUAGGUCAGU
chi-miR-126-5p	F: CAUUAUUACUUUUGGUACGCGC<br>R: GCGCGUACCAAAAGUAAUAAUG	GCGCGUACCAAAAGUAAUAAUG
chi-miR-30a-5p	F: UGUAAACAUCCUCGACUGGAA-GCU<br>R: AGCUUCCAGUCGAGGAU-GUUUACA	AGCUUCCAGUCGAGGAUGUUUACA

Table S3. miRNA qPCR primers.

miRNA	Stem-loop reverse transcription primers (5'→3')	Forward primer (5'→3')	General reverse primer (5'→3')
chi-miR-204-5p	GTCGTATCCAGTG-CAGGGTCCGAGGTATTCGCACTG-GATACGACAAGGCAUA	UUCCCUUUGUCAUCCUAU	AACGCTTCAC-GAATTTGCGT
chi-miR-200a	GTCGTATCCAGTG-CAGGGTCCGAGGTATTCGCACTG-GATACGACTCGATTAC	UAACACUGUCUGGU-AACGA	
chi-miR-192-5p	GTCGTATCCAGTG-CAGGGTCCGAGGTATTCGCACTG-GATACGACGGCTGTC	CUGACCUAUGAAU-UGACAG	
chi-miR-126-5p	GTCGTATCCAGTG-CAGGGTCCGAGGTATTCGCACTG-GATACGACGCGCGTA	CAUUAUUACU-UUUGGUACG	
chi-miR-30a-5p	GTCGTATCCAGTG-CAGGGTCCGAGGTATTCGCACTG-GATACGACAGCUUCC	UGU-AAACAUCCUCGACUGG	
U6	GTCGTATCCAGTG-CAGGGTCCGAGGTATTCGCACTG-GATACGACAAAATATGGAAC	CTCGCTTCGGCAGCACA	

Disclaimer/Publisher's Note: The statements, opinions and data contained in all publications are solely those of the individual author(s) and contributor(s) and not of MDPI and/or the editor(s). MDPI and/or the editor(s) disclaim responsibility for any injury to people or property resulting from any ideas, methods, instructions or products referred to in the content.
